# Machine Learning-Based Identification of Candidate Serum miRNA Features for Pan-Cancer and Cancer Type Classification

**DOI:** 10.3390/life16050850

**Published:** 2026-05-20

**Authors:** Kaiyan Feng, Yusheng Bao, Jingxin Ren, Wei Guo, Deling Wang, Tao Huang, Yu-Dong Cai

**Affiliations:** 1 Department of Computer Science, Guangdong AIB Polytechnic College, Guangzhou 510507, China; kyfeng@gdaib.edu.cn; 2 School of Life Sciences, Shanghai University, Shanghai 200444, China; bys@shu.edu.cn (Y.B.); ssdrg@shu.edu.cn (J.R.); 3 Shenzhen Institute of Advanced Technology, Chinese Academy of Sciences, Shenzhen 518055, China; gw_1992@sjtu.edu.cn; 4 Department of Radiology, State Key Laboratory of Oncology in South China, Guangdong Provincial Clinical Research Center for Cancer, Sun Yat-sen University Cancer Center, Guangzhou 510060, China; wangdl@sysucc.org.cn; 5 Bio-Med Big Data Center, CAS Key Laboratory of Computational Biology, Shanghai Institute of Nutrition and Health, University of Chinese Academy of Sciences, Chinese Academy of Sciences, Shanghai 200031, China; 6 CAS Engineering Laboratory for Nutrition, Department of Artificial Intelligence and Digital Health, Shanghai Institute of Nutrition and Health, University of Chinese Academy of Sciences, Chinese Academy of Sciences, Shanghai 200031, China

**Keywords:** miRNA, pan-cancer, machine learning, candidate classificatory feature

## Abstract

MicroRNA (miRNA) regulation plays a pivotal role in intracellular gene expression. Analysis of miRNA profiles can provide critical insights into disease states. As cancer-associated molecules reported in previous studies, miRNAs may serve as candidate classificatory features for exploratory cancer classification. This research analyzed serum miRNA data from patients with 13 solid cancer types and individuals without cancer. The study comprised two distinct analyses: first, stratifying the dataset into cancer and non-cancer groups to identify miRNAs differentially represented in cancer patients; and second, subdividing the cancer patient data into 13 predefined solid-cancer types to identify candidate miRNA features that discriminate among these cancer types. We employed seven feature-ranking algorithms to evaluate miRNA contributions in both analyses and generate feature lists. Each list was examined using an incremental feature selection method to extract essential miRNAs and build good-performing classification models. Several candidate miRNAs were identified for distinguishing pan-cancer samples from non-cancer ones: miR-4783-3p has been linked to associated with the regulation of endocrine cell differentiation, and miR-663a has been reported in hepatocellular carcinoma and thyroid carcinoma. The analysis also highlighted miRNAs that differentiate solid cancer types, including miR-629-3p, reported to be upregulated in lung and breast cancer, and miR-6087, reported to be downregulated in osteosarcoma and bladder cancer.

## 1. Introduction

Cancer is one of the major diseases worldwide; it is a complex disease that develops largely through genetic alterations in normal cells. These alterations drive uncontrolled cell proliferation and invasion into other tissues and organs. Early-stage cancers are relatively curable, but when cancer metastasizes [1], mortality increases substantially. Tumor metastasis accounts for most cancer-related deaths. Early detection of cancer, therefore, is of utmost importance. Genomic changes vary significantly among various cancers. Similarly, the expression profiles between primary and metastatic tumors are quite dissimilar with considerable variability [2]. Current medical practice still cannot consistently provide early detection or accurate assessment across cancer types. This presents a strong rationale for developing effective predictive methods. Extensive studies have identified microRNAs (miRNAs) as important cancer-associated markers [3,4,5]; miRNAs have been reported to show altered expression in tumor cells and have been investigated as candidate therapeutic targets in prior studies [6].

miRNAs, a family of small non-coding RNAs, typically 20–25 nucleotides in length, are important regulators of gene expression in cells. They function by binding to the mRNA of target genes, inhibiting translation or promoting mRNA degradation, thereby regulating gene expression levels [7,8]. Therefore, miRNAs are involved in cell proliferation, apoptosis, and metabolic processes [9]. They may participate in the pathogenesis of many cancer types, partly because miRNAs regulate metastasis-related factors in several cancer types [10,11]. In particular, Hussen et al. have provided a comprehensive framework illustrating how individual miRNAs can serve simultaneously as signatures of cancer progression, tumor staging, and therapeutic response—functioning as oncogenes in some tissue contexts while acting as tumor suppressors in others [11]. This review further emphasized that the diagnostic and prognostic value of circulating miRNAs depends critically on cancer type, disease stage, and the specific biological pathway involved, cautioning against treating any single miRNA as a universally decisive marker. This framework is directly relevant to the present study because the candidate serum miRNAs identified by our machine learning-based pipeline should be interpreted within the broader, context-dependent landscape of miRNA–cancer interactions documented across multiple independent studies. Depending on cancer type, miRNAs can function as either oncogenes or tumor suppressors. For instance, the miR-24 family facilitates tumor growth in oral cancer but serves a tumor-suppressive role in colorectal cancer [12,13]. Our prior studies also suggested that miRNAs participate in mRNA co-expression networks through gene silencing, a mechanism associated with the onset and progression of testicular germ cell tumors [14]. Moreover, we have identified mRNA-miRNA-lncRNA regulatory networks in ovarian cancer and uterine corpus endometrial carcinoma. In these networks, miRNAs interact with lncRNAs through sponge-like mechanisms, attenuating gene-silencing capacity and potentially contributing to cancer initiation and metastasis [15,16]. These findings indicate the context-dependent roles of miRNAs in tumor regulation through diverse pathways. As candidate molecular features, miRNAs may support disease classification, diagnosis and prognostic assessment [10]. The detection of miRNA levels in bodily fluids offers opportunities for early disease diagnosis and prognostic evaluation.

Compared with traditional tissue biopsies, which remain important for tumor characterization, liquid biopsies are notable for their minimal invasiveness and ease of sampling. The repeatability of liquid biopsies enables more effective monitoring of tumor progression, facilitating early cancer diagnosis and treatment response assessment [17,18]. Current technological advances enable precise detection of circulating nucleic acids in serum [19]. Because of the complexity of serum and transcriptomic data, most modern research relies on machine learning analysis methods. Feature selection can partition large datasets into informative subsets; this greatly simplifies the process of biomarker identification [20]. Moreover, a variety of computational and statistical methods have been utilized to identify cancer biomarkers [21], including the analysis of miRNA based features for assessing breast cancer severity and comprehensive pan-cancer evaluations of genomic profiles [22,23]. Such identification is complicated by the complex biological mechanisms of cancer; studies often identify candidate pan-cancer classificatory features associated with multiple cancer types. Thus, another focus of current research is scoring and ranking candidate features using multi-omics data to further improve the predictive power of the pan-cancer biomarkers [24].

In this study, serum miRNA profiles for various cancer types retrieved from the GEO database were analyzed. The profiles were divided into two datasets. The first dataset contained non-cancer and cancer patients, whereas the second contained only cancer patients, who were further classified into thirteen cancer types. Using seven feature-ranking algorithms, including Least absolute shrinkage and selection operator (LASSO) [25], Light gradient boosting machine (LightGBM) [26], Monte Carlo feature selection (MCFS) [27], Minimum redundancy maximum relevance (mRMR) [28], Random forest (RF_ZL) [29], Categorical boosting (CATboost) [30], and Extreme gradient boosting (XGBoost) [31], we obtained seven feature lists for each dataset. Each feature list was fed into an incremental feature selection (IFS) method [32], using four classification algorithms (Decision tree (DT) [33], K-nearest neighbors (KNN) [34], Random forest (RF) [29], and Support vector machine (SVM) [35]) and Synthetic minority oversampling technique (SMOTE) [36], to build optimal classification models and extract essential features and classification rules. These selected features were considered candidate pan-cancer or cancer type classificatory features. Analysis of these features may clarify serum-based classification patterns and generate hypotheses for subsequent biological validation.

## 2. Materials and Methods

**Figure 1:**
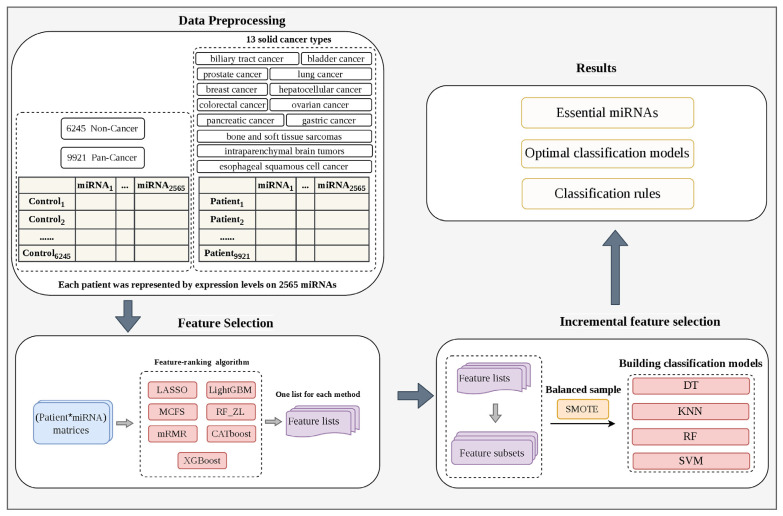
Flow chart of the entire analysis process. Serum miRNA profiles from pan-cancer patients (thirteen solid cancer types) and non-cancer populations were analyzed using multiple machine learning algorithms. Seven feature-ranking algorithms, four classification algorithms, and Synthetic minority oversampling technique (SMOTE) were included. Feature screening was performed using incremental feature selection method, yielding key miRNAs and classification rules, which indicated quantitative miRNA expression patterns for specific populations. Patient*miRNA means the patient-miRNA expression matrices using patients as rows and miRNAs as columns.

This study analyzed serum miRNA profiles from cancer patients and non-cancer individuals. Figure 1 outlines the overall workflow of this study. Initially, these data were converted into two patient miRNA expression matrices, corresponding to cancer versus non-cancer classification and cancer type classification. Each matrix was investigated by seven feature-ranking algorithms, resulting in seven feature lists. Subsequently, the IFS method was used to identify the essential miRNA features from each list and build high-performing classification models.

### 2.1. Dataset

This study used data retrieved from the GEO database with accession ID GSE211692 (https://www.ncbi.nlm.nih.gov/geo/query/acc.cgi?acc=GSE211692, accessed on 20 March 2023) [37], which included serum miRNA profiles from pan-cancer patients with thirteen solid cancer types and non-cancer populations. Two independent analyses were conducted, corresponding to two datasets. First, the patients were divided into one pan-cancer group (9921 patients) and one non-cancer group (6245 patients). Second, the cancer patients were further divided into thirteen groups based on thirteen cancer types, including biliary tract cancer, bladder cancer, bone and soft tissue sarcomas, breast cancer, colorectal cancer, esophageal squamous cell cancer, gastric cancer, hepatocellular cancer, intraparenchymal brain tumors, lung cancer, ovarian cancer, pancreatic cancer, and prostate cancer. Table 1 shows the number of patients in each group. Each patient in the two datasets was represented by the expression levels of 2565 miRNAs. Each dataset was transformed into a two-dimensional matrix (patient-miRNA expression) and fed into a machine-learning-based workflow.

### 2.2. Feature-Ranking Algorithms

To analyze the importance of miRNAs in identifying cancer patients from non-cancer populations or classifying solid cancer types, we employed seven feature-ranking algorithms, including LASSO [25], LightGBM [26], MCFS [27], mRMR [28], RF_ZL [29], CATboost [30], and XGBoost [31]. These algorithms have been widely used in marker-screening studies [38,39,40,41,42]. In this study, they were applied to the two complete datasets for ranking miRNAs based on how well they are associated with the target variable. Brief descriptions of these algorithms are provided in File S1. We used the public packages for these seven algorithms and executed them with their default hyperparameters. The sources of the packages are provided in Table S1.

### 2.3. Incremental Feature Selection

IFS [32] is an approach for selecting important features from a given feature list. First, several feature subsets are constructed such that the top feature in the list constitutes the first feature subset, then the next feature in the list is added to constitute the second feature subset, and so on. Second, one classification model based on a given classification algorithm is constructed on each feature subset, which is evaluated using a cross-validation method [43] and assessed using metrics such as accuracy, F1 measure, or AUC. Finally, the best-performing model is identified as the optimal model, and the features used in this model are extracted as the optimal features. These features generally make essential contributions to classification and should be further analyzed.

### 2.4. Synthetic Minority Oversampling Technique

SMOTE [36] is an algorithm widely used to address class imbalance in machine learning. It is mainly used when the number of majority-class samples in real-world classification tasks far exceeds the number of minority-class samples. The algorithm operates as follows: first, a sample is randomly selected from the minority class, and its nearest *k* neighbors in the same class are identified according to the parameter ‘*k*’ specified by the user; a new sample is then generated by selecting a new point on the line between the selected sample and its randomly selected neighbor. This new sample is assigned to the minority class to increase its size. The process of generating new samples is repeated until the desired balance ratio is reached between the minority classes and the majority classes.

In this study, SMOTE was used to balance the datasets in two analyses. In the first analysis, cancer patients outnumbered non-cancer patients. SMOTE generated new representations of non-cancer patients until the number of non-cancer patients matched the number of cancer patients. In the second analysis, lung cancer patients constituted the largest class. SMOTE produced new representations of patients in other classes until all classes had equal sizes. Importantly, SMOTE was only applied to the training set in each round of cross-validation. This strategy isolated test sample information from the training procedure, making the results more reliable.

### 2.5. Classification Algorithms

A total of four classification algorithms were used in this study to construct classification models in the IFS procedure, including DT [33], KNN [44], RF [29], and SVM [45]. Their brief descriptions are available in File S1. In this study, we used the packages of these four classification algorithms implemented in Scikit-learn 1.2.1 [46]. Each package was run using default hyperparameters.

### 2.6. Cross-Validation Strategy

To ensure the robustness and generalization capability of the classification models in the IFS procedure, we employed a ten-fold cross-validation approach [43,47,48,49,50,51]. The dataset was randomly partitioned into ten equal-sized subsets (folds), maintaining the proportion of samples in each class across all folds. In each iteration, nine folds were used for model training while the remaining fold served as the validation set. This process was repeated ten times, with each fold serving as the validation set exactly once. The final performance metrics were calculated as the average across all ten iterations, providing a more reliable estimate of model performance.

### 2.7. Performance Evaluation

The purpose of the first analysis was to identify candidate miRNA classificatory features that could distinguish pan-cancer patients from the non-cancer population. This is a binary classification problem. F1 measure [52,53,54,55,56,57,58,59,60] was used as the key performance metric. Other metrics, such as sensitivity (SN), specificity (SP), accuracy (ACC), precision, and Matthews correlation coefficient (MCC) [61], were also provided for reference. In the second analysis, the samples (patients) were divided into thirteen cancer types. Because the dataset was imbalanced, weights were introduced when evaluating models built on this dataset. The weighted F1 was selected as the key metric and other metrics, including ACC, MCC, and macro F1, were provided for reference.

## 3. Results

### 3.1. Results of Feature-Ranking Algorithms

After the serum miRNA profiles were converted into two two-dimensional matrices, each matrix was analyzed by seven feature-ranking algorithms independently, yielding seven feature lists, which are provided in Table S2. Typically, miRNAs (referred to as features in this study) with the highest relevance to cancer were generally positioned at the top of these lists. In the following analysis, we focused on the top features in each list.

### 3.2. Results of IFS

Each feature list mentioned in Section 3.1 was fed into IFS for subsequent analysis. Generally, essential miRNAs for distinguishing cancer patients from non-cancer individuals and classifying cancer types are usually limited in number. Thus, we only considered the top 1000 features in each list. The inclusion of more features may introduce noise, influencing the reliability of the results. Furthermore, we used a step size of five to construct feature subsets to accelerate the IFS procedure due to our limited computing resources, resulting in 200 subsets from each list. On each subset, four classification models using DT, KNN, RF, and SVM as classification algorithms were constructed and evaluated by ten-fold cross-validation. The cross-validation results were summarized as the metrics mentioned in Section 2.7. In detail, SN, SP, ACC, MCC, precision, and F1 measure were calculated in the first analysis, which are summarized in Table S3, whereas the ACC, MCC, macro F1, and weighted F1 were computed in the second analysis, which are presented in Table S4. The F1 measure was selected as the key metric in the first analysis. Accordingly, IFS curves were plotted to show the dynamics of the model performance as the number of features changed, where F1 measure and number of features were set as the *y*-axis and *x*-axis, respectively. The IFS curves for different classification algorithms on seven feature lists are provided in Figures S1 and S2. Likewise, the IFS curves for the second analysis are provided in Figures S3 and S4, where weighted F1 and number of features were set as the *y*-axis and *x*-axis, respectively. The results show that the number of features used and predictive ability were not linearly related. There was a significant performance gap between the classification models constructed using different classification algorithms on different feature lists.

For the first analysis on pan-cancer patients and non-cancer populations, the IFS procedure identified the optimal models on seven feature lists. Specifically, on the feature list yielded by mRMR, the DT model achieved the peak performance with an F1 measure of 0.956, using the top 175 features. The KNN model performed the best on three lists yielded by CATboost, LASSO, and LightGBM. The F1 measures of these models were 0.977 on the feature list yielded by CATboost with the top 50 features, and 0.962 and 0.979 on feature lists yielded by LASSO and LightGBM, respectively, with the top 25 miRNAs each. As for the feature lists yielded by MCFS, RF, and XGBoost, the RF models performed the best. They yielded the F1 measures of 0.962, 0.966, and 0.970 with the top 645 features in the list yielded by MCFS, the top 995 features in the list yielded by RF_ZL, and the top 35 features in the list yielded by XGBoost, respectively. The detailed performance of the above optimal models is listed in Table 2. Specifically, the KNN model using the top 25 features in the list yielded by LightGBM provided the highest F1 measure. This model produced balanced performance, with SN of 0.985, SP of 0.983, and precision of 0.973. It may serve as a useful tool for distinguishing cancer patients from non-cancer individuals in this dataset.

For the second analysis on a cancer patient dataset, the aim was to identify miRNAs that could distinguish thirteen different cancer types. The optimal models on different feature lists were also identified through the IFS procedure and evaluated using weighted F1. This time, the optimal models all used SVM as the classification algorithm across seven feature lists. In particular, they used the top 155, 195, 245, 480, 615, 295, and 240 features in the lists yielded by CATboost, LASSO, LightGBM, MCFS, mRMR, RF_ZL, and XGBoost, respectively, and yielded weighted F1 scores of 0.878, 0.795, 0.884, 0.869, 0.850, 0.876, and 0.832, respectively. The detailed performance of the above optimal models is provided in Table 3. Among these, the SVM model using the top 245 features in the list yielded by LightGBM provided the best performance. The performance of this model on thirteen cancer types, measured by F1 measure, is listed in Table 4. Eight cancer types had F1 measures higher than 0.9, and one cancer type had an F1 measure lower than 0.8, indicating high and balanced performance of the model. These results suggest that this model may be useful for identifying different cancer types.

### 3.3. Uncovering Biologically Significant Candidate miRNAs

**Figure 2:**
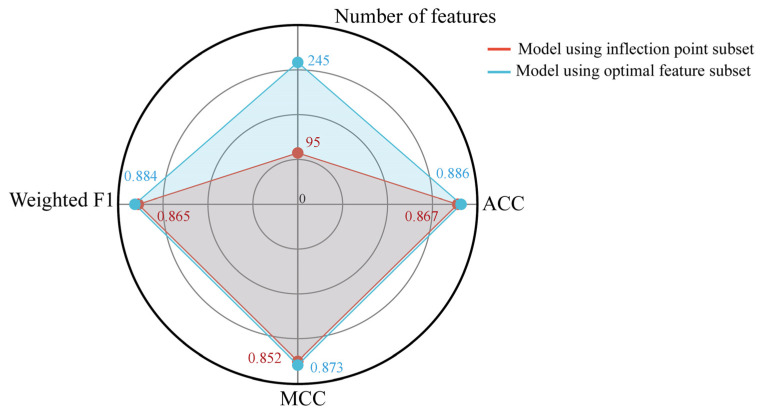
Radar plot to show the performance of the classification models using the optimal feature and inflection point subsets on the feature list yielded by LightGBM in the second analysis on thirteen cancer types. Radar plot showed the three metrics of two models (ACC, MCC, weighted F1) and the number of features used. The model using inflection point subset yielded similar performance but need much fewer features.

Through the IFS procedure, we obtained several optimal models and features from the two analyses. The optimal features may represent informative serum miRNAs for identifying pan-cancer patients or different cancer types, thereby providing candidate classificatory signals for further evaluation. However, several optimal models used many optimal features, which may include false-positive features. In view of this, we looked for the inflection points in the IFS curves containing the highest F1 measure or weighted F1 yielded by the optimal models using more than 100 features. The inflection point was determined by the threshold-based method. The threshold for F1 measure or weighted F1 was empirically selected according to the trends of the IFS curves. The point first exceeding the threshold was defined as the inflection point. The features corresponding to the inflection point constituted the inflection point subset. The performance of the models using inflection point subsets is listed in Table 2 and Table 3. Their performance was slightly lower than that of the optimal models but required far fewer features. Figure 2 shows the comparison between models using the optimal feature subset and the inflection point subset on the list yielded by LightGBM for the second analysis. The ACC, MCC, and weighted F1 were slightly reduced, whereas the number of features was reduced from 245 to 95. This suggested that the inflection point subset retained the most informative features from the optimal feature set.

**Figure 3:**
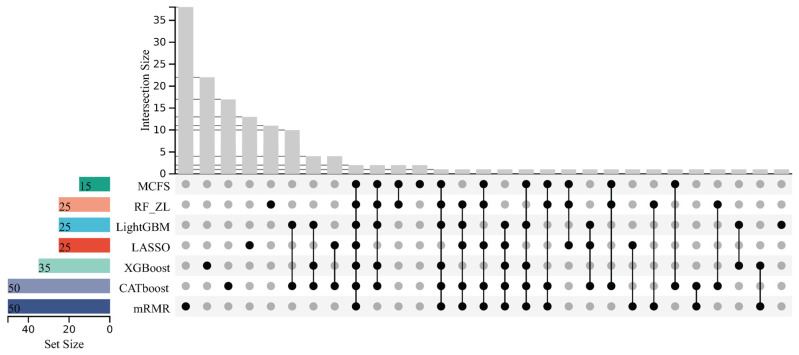
UpSet graph of the inflection point subsets extracted from the lists yielded by LASSO, LightGBM, MCFS, mRMR, RF_ZL, CATboost and XGBoost for the analysis on pan-cancer and non-cancer populations. The graph was plotted based on the number of occurrences of features and the subsets in which they were located. The black dots represented the subsets where the features were located.

**Figure 4:**
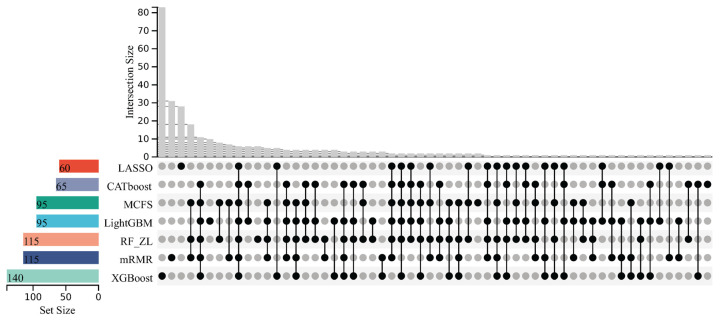
UpSet graph of the inflection point subsets extracted from the lists yielded by LASSO, LightGBM, MCFS, mRMR, RF_ZL, CATboost and XGBoost for the analysis on thirteen cancer types. The graph was plotted based on the number of occurrences of features and the subsets in which they were located. The black dots represented the subsets where the features were located.

To compare the inflection point subsets extracted from different feature lists, an UpSet graph was plotted for each analysis, as shown in Figure 3 and Figure 4. When the optimal feature subset contained fewer than 100 features, we did not identify an inflection point or a corresponding inflection point subset. The optimal feature subset was then treated as the inflection point subset for consistency. The detailed features occurring in different numbers of inflection point subsets are provided in Table S5. It can be observed that several features occurred in multiple inflection point subsets, indicating that the corresponding miRNAs were identified as essential by multiple feature-ranking algorithms. These miRNAs therefore warrant further investigation as candidate classificatory features.

### 3.4. Quantitative Characterization of miRNA Expression Patterns in Different Populations

Among the four classification algorithms used in this study, DT differs from the other three algorithms. It is a white-box algorithm, providing transparent classification procedures. This provides further insight into the essential differences encoded in the two datasets between pan-cancer and non-cancer patients and among patients with different cancer types. We selected the optimal features for DT on each feature list and used all patients to train the tree. A group of classification rules was obtained from the tree and is provided in Tables S6 and S7. Each rule contains several features, their thresholds, and one result (class: non-cancer or pan-cancer in the first analysis and one of thirteen cancer types in the second analysis). Because the features represent miRNAs, each rule indicates a specific miRNA expression pattern for a class. The obtained rules can be useful resources for a deeper investigation into the molecular-level alterations in cancer patients and may help generate hypotheses about associated biological processes that warrant further investigation.

## 4. Discussion

Using seven feature-ranking algorithms, we identified a set of key miRNAs associated with cancer status and cancer type in this serum dataset. These miRNAs contributed to classification by helping differentiate cancerous from non-cancerous states and distinguish cancer types. This approach nominates candidate serum miRNA features that warrant further investigation as classificatory signals.

To ensure that the biological interpretation of these miRNAs is anchored in the present dataset rather than in prior literature alone, for each identified miRNA we first characterize its behavior within our serum cohort, including the direction and magnitude of change between cancer and non-cancer groups, class-specific distribution across thirteen solid cancer types, and statistical significance after multiple-testing correction, and only then relate these observations to previously reported functions. In the discussion that follows, prior literature is used to contextualize, not to substitute for, the evidence provided by the present study.

### 4.1. Feature Analysis for Cancer Versus Non-Cancer Identification

**Figure 5:**
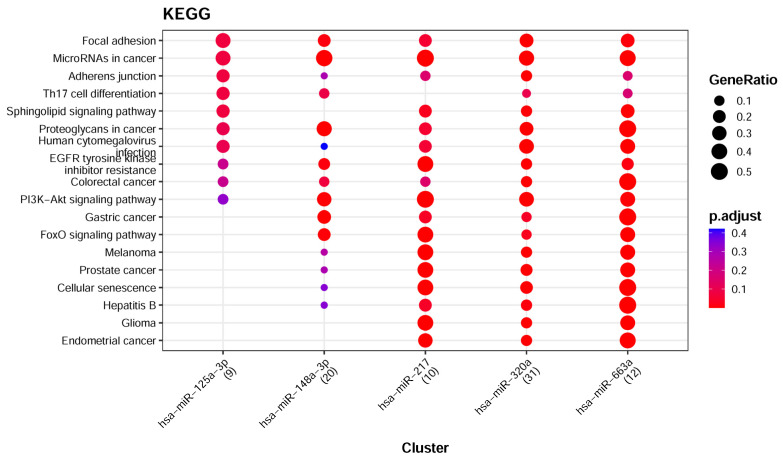
KEGG functional enrichment analysis of the top 5 miRNAs with the highest degree (Pan-Cancer vs. non-Cancer).

**Figure 6:**
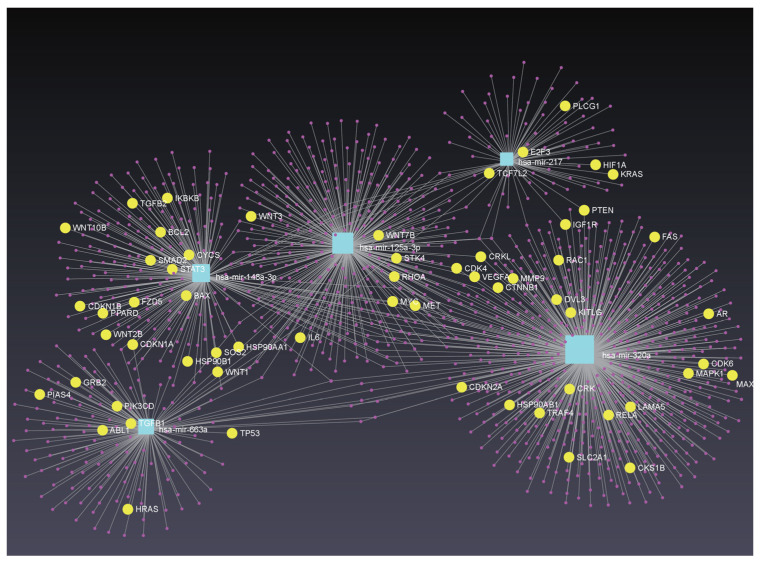
miRNA–Target interactions network of the top 5 miRNAs and KEGG functional enrichment analysis (Pan-Cancer vs. non-Cancer). The yellow dots represent the genes related to Pathways in cancer. A concise functional annotation of these 59 genes, together with the top 5 miRNAs that regulate each of them, is provided in Table S9.

The seven feature-ranking algorithms collectively identified 144 key miRNAs (Table S5), of which 137 miRNAs were mapped using the Mienturnet platform and the miRTarBase database [62]. To focus on miRNA–target interactions, the top 5 miRNAs with the highest node counts were selected for KEGG enrichment analysis, as detailed in Figure S5. As shown in Figure 5, the focal adhesion and adherens junction pathways are significantly enriched. These pathways are involved in cellular motility and migration through the extracellular matrix, and altered pathway activity has been associated with tumor metastasis and drug resistance. Therefore, they provide relevant biological context for interpreting the selected serum miRNA features [63,64,65]. These observations are consistent with our previous KEGG analyses of cancer gene expression profiles, which also implicated cancer-related pathways [66,67]. Moreover, the enrichment of “MicroRNAs in cancer” is consistent with the classificatory signal observed in our serum data, but should be interpreted as hypothesis-generating rather than as evidence of direct tissue-level mechanism. The enrichment analysis also highlighted signaling pathways related to specific cancer types, providing context for cancer type discrimination. Furthermore, a network containing miRNA–target interactions for these five miRNAs was constructed using the miRNet platform (Figure 6), identifying 1250 gene targets [68]. Further KEGG enrichment analysis revealed that the “Pathways in Cancer” pathway was the most enriched (Table S8), with corresponding genes represented by yellow dots inside the network. Notably, some of these genes overlapped with biomarkers reported in our previous cancer studies, providing context for the current findings [66].

To annotate the cancer-related context of the highlighted yellow nodes, we grouped the 59 “Pathways in cancer” genes by functional module. They span the canonical subprograms of KEGG hsa05200: cell cycle and proliferation control (*MYC*, *MAX*, *CDK4/6*, *CDKN1A/1B/2A*, *E2F3*, *CKS1B*); PI3K–AKT and RAS–MAPK signaling downstream of receptor tyrosine kinases (*HRAS*, *KRAS*, *MAPK1*, *GRB2*, *SOS2*, *PIK3CD*, *CRK/CRKL*, *IGF1R*, *MET*, *KITLG*); the Wnt/β-catenin axis (*WNT1/2B/3/7B/10B*, *FZD5*, *DVL3*, *CTNNB1*, *TCF7L2*); TGF-β/SMAD signaling (*TGFB1/2*, *SMAD2*); apoptosis regulation (*TP53*, *BCL2*, *BAX*, *CYCS*, *FAS*); tumor suppression and Hippo/SUMO regulation (*PTEN*, *STK4*, *PIAS4*); inflammation and cytokine signaling (*IL6*, *STAT3*, *RELA*, *IKBKB*, *TRAF4*); angiogenesis and invasion (*VEGFA*, *HIF1A*, *MMP9*, *LAMA5*, *RHOA*, *RAC1*); and additional cancer-associated effectors including nuclear receptors (*AR*, *PPARD*), the oncogenic tyrosine kinase ABL1, HSP90 chaperones (*HSP90AA1/AB1/B1*), the HIF-regulated glucose transporter SLC2A1 (Warburg effect metabolism), and *PLCG1*. A concise per-gene annotation with the top-5 regulating miRNA(s) is provided in Table S9. These miRNA–target interactions provide pathway-level context for the serum miRNA features.

Before relating these selected features to the existing literature, we first summarize their behavior within our own serum cohort (Table S10; class-specific distributions are shown in Figure S6). All features highlighted in this section showed significant differential expression between pan-cancer and non-cancer samples by two-sided Wilcoxon rank-sum test with Benjamini–Hochberg correction (q < 0.005 in every case). Specifically, miR-4783-3p (log2FC = +2.63; median log2 expression increased from 5.34 in non-cancer to 8.54 in pan-cancer; selected by 7/7 ranking methods), miR-663a (log2FC = +1.66; 10.51 to 12.30; 7/7), miR-5100 (log2FC = +3.08; 10.22  to  13.73; 5/7), miR-1307-3p (log2FC = +2.52; 5.47  to  8.42; 5/7), miR-8073 (log2FC = +2.32; 5.69  to  8.27; 4/7), miR-1469 (log2FC = +1.27; 10.58  to  11.91; 2/7) and miR-151a-5p (log2FC = +4.54; −2.95  to  3.06; 1/7) were markedly upregulated in pan-cancer serum relative to non-cancer controls, whereas miR-6784-5p (log2FC = −1.27; 12.64 to 11.26; 6/7) and miR-4456 (log2FC = −0.45; 2.19  to  0.34; 3/7) were downregulated. These quantitative observations, drawn from the present cohort, serve as the primary basis for the candidate classificatory feature claims made below; the cited literature is intended to place these findings in biological context, not to substitute for them.

Within the 144 identified key features, the highest-ranked features contributed most to discriminating cancerous from non-cancerous states, leading us to select certain features for in-depth analysis grounded in the existing literature. miR-4783-3p, recognized by all seven feature-ranking algorithms, was retained as a candidate classificatory feature in this serum dataset. Previous research has shown that it targets the *INSM1/IA-1* gene [69], which is linked to insulinoma and is crucial for endocrine cell differentiation, marking miR-4783-3p as a potential indicator of endocrine differentiation in various tumors [70,71]. Moreover, miR-4783-3p has been acknowledged for its high diagnostic accuracy as a biomarker for ovarian cancer [69]. Similarly, miR-663a, identified by all seven algorithms, has been reported across several cancer types; prior tissue-based studies described its candidate function as a tumor suppressor through TGF-β1 regulation in liver [72] and thyroid cancer [73] cells. The regulatory elements of TGF-β signal transduction, including the pan-cancer biomarker Smad protein we previously discovered, are prevalent in numerous cancers [74]. miR-663a also inhibits the proliferation and invasion of glioblastoma cells by targeting *PIK3CD* and acts as a tumor suppressor in gastric and lung cancers [75,76,77]. Thus, miR-4783-3p and miR-663a are highlighted by our analysis as key candidate classificatory features warranting further validation.

For instance, the presence of miR-6784-5p in six feature subsets reflects its dysregulation in several cancers such as ovarian cancer [78], breast cancer [79], and prostate cancer [80]. It is therefore a candidate classificatory feature for further evaluation [81]. miR-1307-3p appears in five feature subsets, suggesting strong relevance to multiple cancers. It targets the DAB2-interacting protein, enhancing proliferation, invasion, and migration of liver cancer cells [82]. Additionally, it influences breast cancer proliferation by targeting the tumor suppressor gene *SMYD4* (SET and MYND domain containing 4) and contributes to gastric and colon cancer development [83,84,85].

miR-8073, found in four feature subsets, is associated with potential targets including *FOXM1*, *CCND1*, *MBD3*, *KLK10*, and *CASP2*, which are crucial in cell cycle regulation and proliferation [86,87,88,89]. In prior tissue-based studies, this miRNA has been reported to act as a candidate tumor suppressor associated with reduced target protein levels and inhibition of tumor growth.

miR-4456, identified in three subsets, shows downregulation in osteosarcoma and acts as a regulatory molecule in metastasis, exhibiting tumor-suppressive properties [90]. miR-1469, present in two subsets, targets *STAT5A* to regulate apoptosis in lung cancer cells [91]. miR-151a-5p, appearing in a single subset, facilitates the epithelial–mesenchymal transition in colorectal cancer cells, similar to the role of *CTBP2*, a colon cancer biomarker we previously identified [92], consistent with the reported role of EMT in colon cancer described in prior tissue-based studies.

### 4.2. Feature Analysis for Cancer Type Classification

**Figure 7:**
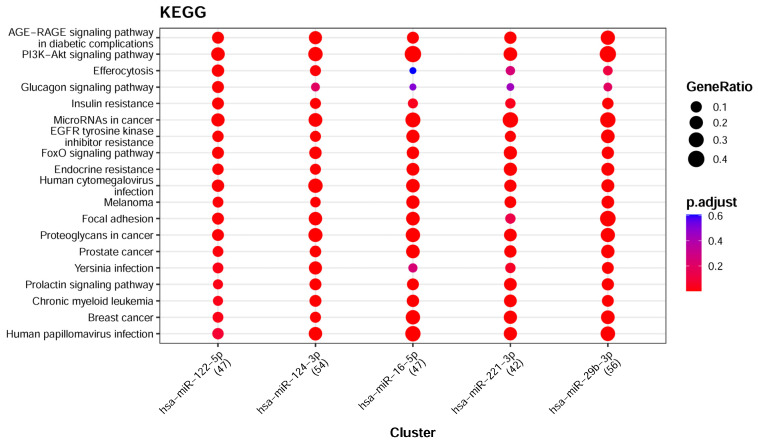
KEGG functional enrichment analysis of the top 5 miRNAs with the highest degree (Within Pan-Cancer).

Seven feature-ranking algorithms identified a total of 307 key miRNAs (Table S5), of which 297 miRNAs were mapped using the Mienturnet platform and the miRTarBase database. To further investigate miRNA-target gene relationships, the top 5 miRNAs were selected for detailed enrichment analysis, as presented in Figure S7. The subsequent KEGG pathway analysis, shown in Figure 7, highlighted several processes that are closely related to the development and progression of cancer. Of particular interest was the involvement of the PI3K-Akt signaling pathway in tumor cell proliferation [93], while the process of Efferocytosis has been noted not only for the engulfment of apoptotic cells but also for modulating the tumor microenvironment to foster immune suppression and promote tumor invasion [94].

**Figure 8:**
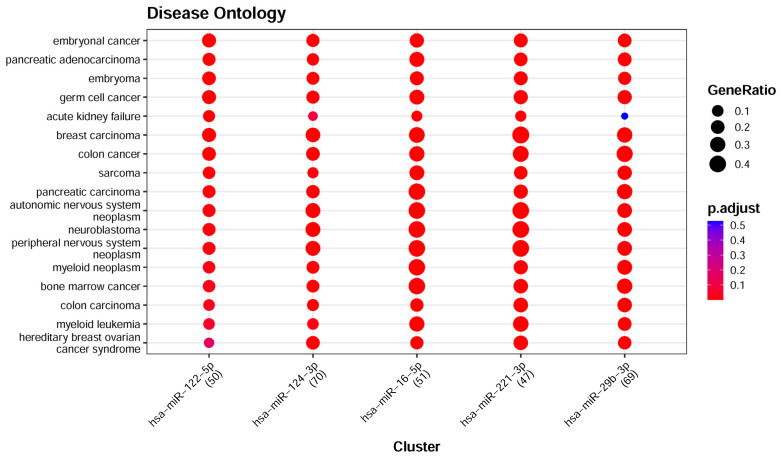
Disease ontology enrichment analysis of the top 5 miRNAs.

Pathways such as MicroRNAs in cancer, Prostate cancer, and Breast cancer, which were highlighted in the enrichment analysis, indicate that miRNAs are useful in the discrimination of cancer types. The application of Disease Ontology enrichment analysis provided additional detail on the association of the miRNAs with different types of cancers. The major disease classes were cancers or tumors; as shown in Figure 8, these classes significantly matched our samples and are consistent with the cancer-type-related signal captured by the selected serum miRNA features within this dataset.

**Figure 9:**
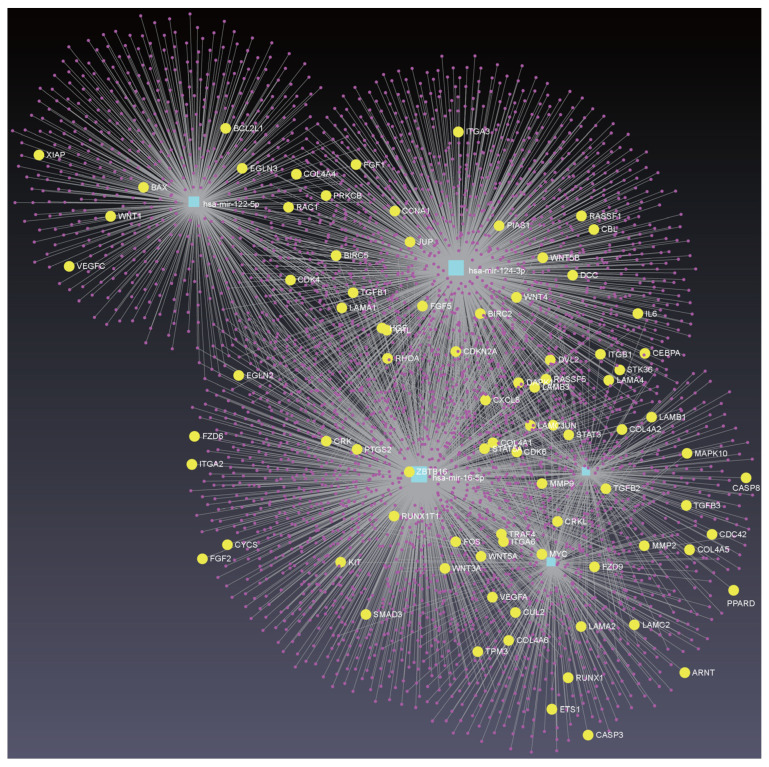
miRNA–Target interaction network of the top 5 miRNAs and KEGG functional enrichment analysis (Within Pan-Cancer).

Meanwhile, the miRNA–target interaction network for the top 5 features was constructed (Figure 9). KEGG pathway analysis indicated that “Pathways in cancer” was the most significantly enriched pathway (Table S11). Notably, several related target genes overlapped with previously identified pan-cancer biomarkers, consistent with previously reported observations.

To examine whether multiple miRNAs in this top-5 set share common targets, we quantified, for each of the 3825 unique genes in the Figure 9 network, how many of the five miRNAs regulate it. Fifty-seven genes are cooperatively targeted by three or more of the five miRNAs as shown in Table S12: one by all five, five by four, and 51 by three. The single gene regulated by all five miRNAs is *AKT3*, one of the three AKT isoforms and a central effector of PI3K–AKT survival and proliferation signaling, whose amplification or overexpression has been reported in ovarian, breast, and glioblastoma cancers and associated with resistance to targeted therapy [95]. Five further genes are each targeted by four of the five miRNAs. *CDK6* is a cyclin-D partner that phosphorylates Rb to drive the G1–S transition and is deregulated in lymphomas, gliomas, melanoma, and breast cancer; it is the clinical target of palbociclib, ribociclib and abemaciclib [96]. *PSMB5* encodes the catalytic chymotrypsin-like β5 subunit of the 20S proteasome, the direct target of bortezomib and carfilzomib in multiple myeloma, and its upregulation or mutation is a recognized mechanism of proteasome inhibitor resistance [97]. *DDX6* is a DEAD-box RNA helicase that regulates mRNA decay and translational repression in P-bodies and is oncogenic in colorectal, hepatocellular and head-and-neck cancers [98]. *GALNT3* (polypeptide N-acetylgalactosaminyltransferase 3) catalyzes initial mucin-type O-glycosylation of Ser/Thr residues and, when aberrantly expressed, contributes to pancreatic, ovarian, and gastric tumor invasion and poor prognosis [99]. *CALU* encodes calumenin, an ER Ca2+-binding chaperone that is upregulated in lung adenocarcinoma and hepatocellular carcinoma, where it has been linked to migration, chemoresistance and epithelial–mesenchymal transition [100]. The 51 genes targeted by three of the five miRNAs include canonical cancer regulators such as *TP53*, *MYCN*, *CCND2*, *PIK3R1*, *MKI67*, *HMGA1* and *SP1*; EMT and invasion/MMP-regulatory factors (*ZEB2*, *RHOA*, *RECK*, *TIMP3*); basement membrane components (*COL4A1/A2*, *LAMC1*); inflammation and immune modulators (*REL/NF-κB*, *ENTPD1/CD39*); the RNAi effector AGO2; and ER stress/chaperone genes (*CALR*, *HMOX1*, *SERPINH1*). Collectively, the cooperatively regulated targets converge on PI3K–AKT signaling, cell cycle control, and ECM/invasion programs. A full annotation of all 57 shared targets, with their regulating miRNA(s) and cancer relevance notes, is provided in Table S12. As with the enrichment results above, this shared-target signal should be interpreted as hypothesis-generating rather than as direct evidence of tissue-level mechanism.

For the cancer-type classification analysis, we again first describe what is directly observable in our own dataset before turning to previously reported functions (Table S11; cross-cancer distributions and the corresponding heatmap are shown in Figure S8). All features highlighted in this section showed highly significant differential expression across the 13 solid tumor types and between pan-cancer and non-cancer samples (BH-FDR q < 0.005 in every case). In aggregate pan-cancer vs. non-cancer comparisons, miR-629-3p (log2FC = +0.21; selected by 7/7 ranking methods in the cancer type analysis), miR-6087 (log2FC = +0.37; 6/7), miR-422a (log2FC = +0.10; 6/7), miR-221-3p (log2FC = +2.61; 5/7), miR-29b-3p (log2FC = +4.07; 4/7), miR-4726-5p (log2FC = +0.29; 3/7) and miR-6165 (log2FC = +0.13; 2/7) were upregulated, while miR-133b (log2FC = −0.32; 1/7) was modestly downregulated. More importantly for cancer type discrimination, several of these features—notably miR-629-3p, miR-221-3p and miR-29b-3p—displayed strongly class-specific distribution patterns across the 13 tumor types (Figure S8), which is the feature-level property that makes them informative for type classification rather than for simple detection of cancer status. The biological discussion that follows should therefore be read as placing these cohort-level observations in the context of prior molecular studies.

Within the 307 identified key features, the highest-ranked features contributed most to discriminating cancer types, leading us to select certain features for in-depth analysis grounded in the existing literature. miR-629-3p, identified in seven feature subsets, emerges as a candidate classificatory feature; tissue-based studies have previously reported its involvement in various cancer types. It has been implicated in cancer progression; for instance, in breast cancer, miR-629-3p targets the leukemia inhibitory factor receptor (*LIFR*), facilitating lung metastasis [101]. In lung cancer, miR-629-3p is upregulated and targets the surfactant protein C (*SFTPC*) to promote tumor cell proliferation [102]. In the case of prostate cancer, it targets large tumor suppressor 2 (*LATS2*) to drive tumor cell development [103], while in the case of pancreatic cancer, it targets FOX3, resulting in the latter’s enhanced tumor-promoting effect [104]. These reports of increased miR-629-3p expression in lung, breast, prostate, and pancreatic cancer tissues support its candidacy as a classificatory feature for these cancer types.

miR-6087, appearing in six feature subsets, is linked to osteosarcoma. Its downregulation, mediated by exosomes from human bone marrow mesenchymal stem cells (hBMSCs), impedes osteosarcoma growth [105]. It has also been identified as a specific candidate classificatory feature for bladder cancer [106]. In both osteosarcoma and bladder cancer, miR-6087’s expression is diminished, reflecting its tumor-suppressive function and marking it as a candidate classificatory feature.

miR-422a, present in six feature subsets, has been implicated across several cancers. It targets *MAPK1* and *AKT1* in colorectal tumors, and proline-rich protein 2 in breast cancer stem cells, inhibiting tumor development [107,108]. In osteosarcoma, miR-422a directly targets *TGFβ2* to regulate downstream transcriptional factors [109] or targets *BCL2L2* and *KRAS*, acting as a tumor suppressor by inhibiting cell proliferation and migration [110]. Furthermore, it is associated with increased chemotherapy sensitivity in gastric cancer [111]. The downregulation of miR-422a in rectal cancer, osteosarcoma, breast cancer, and gastric cancer tissues highlights its significance as a candidate classificatory feature.

miR-221-3p, expressed in five subsets, promotes proliferation and inhibits apoptosis in pancreatic cancer cells [112], and impedes ovarian cancer progression and migration by targeting *ARF4* [113]. It has also been reported as a diagnostic and prognostic marker for colorectal and gastric cancers [114,115]. Its involvement in the regulatory network of hepatocellular carcinoma is also substantial [116]. Therefore, miR-221-3p has been reported to be highly expressed in these cancers and is considered a critical candidate classificatory feature for pancreatic, ovarian, colorectal, liver, and gastric cancers.

miR-29b-3p, expressed in four subsets, has been described as a crucial regulatory molecule in the process of epithelial-to-mesenchymal transition in bladder and colorectal cancers [117,118]. Moreover, it plays a significant role in the treatment of breast and ovarian cancers [118,119], and it has been described as an important diagnostic marker in prostate cancer [120]. Accordingly, given its reported downregulation in these cancers, miR-29b-3p may serve as a candidate classificatory feature for these conditions.

miR-4726-5p, expressed in three subsets, acts as a tumor suppressor in hepatocellular carcinoma [121]; it was documented to be downregulated in that cancer type and may thus be considered a candidate feature for that carcinoma. miR-6165 is expressed in two subsets and induces proliferation and migration in hepatocellular carcinoma, as well as in breast cancer [122,123], and its upregulation in these tissues marks it as a candidate feature for these cancers. Finally, miR-133b appeared in one subset and is reported as a tumor suppressor in esophageal cancer [124], whose downregulated expression marks its potential as a candidate classificatory feature for the same type of cancer.

In summary, supported by multiple research studies, the selected features represent promising candidate signatures that warrant further validation in independent cohorts.

### 4.3. Cancer Versus Non-Cancer Classification Rule Analysis

Among the features identified by the seven classifiers, miR-663a and miR-5100 are especially strong candidate classificatory features in this dataset. As described above, miR-663a expression is upregulated in a wide range of cancers, suggesting that its candidate classificatory potential is broad. Similarly, miR-5100 expression is also upregulated in tumor cells, and overexpression of miR-5100 has been shown to increase the proliferation of lung cancer cells and downregulate Rab6 expression, leading to the growth and metastasis of tumors [125]. Conversely, miR-5100, overexpressed in pancreatic cancer, suppresses cell proliferation, migration, and invasion [126]. It is also identified as a critical regulator of apoptosis in gastric cancer [127] and a detection marker in prostate cancer [128]. While miR-4730 was selected in six classifier subsets and has previously been described in pancreatic cancer literature as a candidate molecular feature, it was reported to inhibit tumor growth in liver cancer [129]. miR-1290 is present in five classifier sets and has been widely described as a candidate classificatory feature in several types of cancers, with its possible application in early diagnosis [130]. Similarly, miR-4456 has also appeared in five classification rule sets, given its association with metastasis suppression in osteosarcoma [90].

In summary, this literature review indicates that the features identified by our machine learning approach nominate candidate miRNA classificatory features for further external validation.

### 4.4. Cancer Type Classification Rule Analysis

miR-1343-3p is consistently present in all 13 pan-cancer classification rules. Therefore, it may act as a candidate classificatory feature in those cancers. Previous studies have identified that miR-1343-3p targets the *TEAD4* gene, thereby regulating its expression and acting as a tumor suppressor in gastric and colorectal cancers [131,132]. In pancreatic and ovarian cancers, miR-1343-3p was inhibited by different lncRNAs, which promote tumor development [133,134]. In brief, miR-1343-3p exhibited striking variations among all 13 cancer types. Among these, established roles in six cancer types have been supported by multiple studies, while the remaining seven cancer types remained unexplored. This finding suggests its potential utility as a candidate classificatory feature.

Our findings suggest that miR-629-3p was selected as a candidate classificatory feature for cholangiocarcinoma, hepatocellular carcinoma, and pancreatic cancer, based on our classification criteria. While the involvement of miR-629-3p in pancreatic and liver cancers has been documented, its role and underlying mechanisms in cholangiocarcinoma warrant further exploration. miR-122-5p was predominantly selected as a candidate classificatory feature for bladder cancer, where urothelial carcinoma-associated 1 (*UCA1*), a marker specific to bladder cancer, is notably increased in bladder tumor cells. Acting as a tumor suppressor [135], miR-122-5p is antagonized by *UCA1*, which sponges miR-122-5p to facilitate tumorigenesis [136], providing biological context for the appearance of miR-122-5p as a candidate classificatory feature in this dataset. miR-187-5p is identified predominantly as a candidate classificatory feature for osteosarcoma and ovarian cancer; in osteosarcoma, it suppresses the *S100A4* gene, inhibiting cell proliferation and metastasis [137], while in ovarian cancer, it targets *DAB2* (homolog-2), curbing epithelial–mesenchymal transition and tumor growth [138]. miR-1225-3p, distinctively associated with a subset of breast cancers, may act as a tumor suppressor, given its reduced expression in tumors. This miRNA is diminished by circ_0000518, which indirectly elevates *SOX4*, thereby enhancing breast cancer cell proliferation and migration [139]. In colorectal cancer, miR-6746-5p, significantly overexpressed, appears to promote metastasis and is important in modulating drug response [140]. For esophageal cancer, miR-3648, which is markedly increased, targets *APC2*, playing a pivotal role in cell proliferation and prognosis [141,142]. miR-221-3p, associated mainly with hepatocellular carcinoma in this analysis, has been reported in prior tissue-based studies to promote tumor growth and invasion through suppression of inhibitors such as *SOCS3* [116,143]. Similarly, miR-328-5p, an oncogene in liver cancer, targets *DAB2IP* to enhance malignancy in hepatocellular carcinoma [144]. miR-4763-3p is mainly a classificatory feature of glioma, bone and soft tissue sarcoma, esophageal cancer, and prostate cancer. Its relationship with these cancers has not been validated, but the expression of this miRNA is upregulated in glioma patients and can be used to distinguish diffuse glioma from non-cancerous conditions [145]. miR-195-5p, a pancreatic cancer candidate classificatory feature, is suppressed in tumors, potentially acting as a tumor suppressor. This suppression is attributed to the B-RAF oncogenic kinase mutation that activates non-coding RNAs, inhibiting miR-195-5p, which is also regulated by various non-coding RNAs in pancreatic tumor progression [146,147]. Lastly, miR-1290, a marker for prostate cancer, is reduced in tumors; its secretion by cancer-associated fibroblasts promotes tumor growth and metastasis [148].

In conclusion, our research has identified candidate classificatory features that exhibit strong correlations with specific cancer types, supporting the internal utility of our classification model and rules as a starting point for further external validation in independent cohorts.

### 4.5. Limitations

Several limitations should be acknowledged. First, all performance metrics were obtained by ten-fold cross-validation on a single serum miRNA dataset without independent external or prospective validation; therefore, internal cross-validation, even when carefully performed, should not be regarded as a substitute for true external prospective validation or as evidence of clinically deployable performance. Accordingly, the selected miRNAs should be interpreted as candidate classificatory features and not as validated clinical biomarkers. The absence of detailed clinical information for the non-cancer cohort also means that benign, inflammatory, or other systemic conditions in some controls could not be fully assessed, which may influence estimates of classificatory specificity. Second, although SMOTE mitigated class imbalance, synthetic oversampling cannot fully substitute for real minority-class samples, and performance on rare cancer types should be interpreted with caution. Third, the biological interpretation of the selected miRNAs relies on prior literature to contextualize our data-driven observations; no direct functional validation was performed. Fourth, circulating miRNA levels are sensitive to pre-analytical and biological factors—including hemolysis, freeze–thaw cycles, storage, fasting, age, sex, stage, treatment, and batch effects—and cohort composition is inherently uneven across tumor types (e.g., breast and ovarian samples are obligately female, prostate male, and sarcoma patients substantially younger). Although all samples originated from a single biobank profiled on a uniform 3D-Gene platform with standard normalization, and Matsuzaki reported no significant association of diagnostic performance with age or sex, sample-level metadata on hemolysis, storage, freeze–thaw history, treatment, and batch were unavailable to us as secondary users. None of the miRNAs highlighted in Section 4.1 and Section 4.2 belongs to well-known hemolysis-sensitive species (e.g., miR-451a, miR-486-5p, miR-92a), making hemolysis-driven confounding less likely to explain the selected features; nevertheless, we cannot formally exclude that part of the classificatory signal reflects demographic or technical structure. Independent validation in prospective, multi-center cohorts with harmonized pre-analytical protocols, matched clinical metadata, and dedicated functional assays will therefore be essential before any candidate miRNA identified here can be considered a validated clinical biomarker.

## 5. Conclusions

Serum miRNA profiles from patients with thirteen solid cancer types (pan-cancer) and non-cancer populations were analyzed using a machine learning approach. The study consisted of two independent analyses: (1) to differentiate between pan-cancer and non-cancer populations, and (2) to classify samples among thirteen predefined solid cancer types within this dataset. For the first analysis, the optimal model showed high internal cross-validation performance for binary classification, and decision-tree-derived rules revealed quantitative miRNA expression thresholds associated with the cancer versus non-cancer distinction. For the second analysis, internal cross-validation-based discrimination among the thirteen cancer types was achieved, and class-specific candidate miRNAs were identified. We derived quantitative classification rules that help summarize serum miRNA expression patterns. It should be noted that this closed-set classification task does not equate to cancer-of-unknown-primary diagnosis or metastatic tumor origin tracing, which would require fundamentally different study designs. These results nominate candidate serum miRNA classificatory features for further investigation rather than validated clinical biomarkers; all findings are based on internal cross-validation of a single retrospective dataset and should be considered exploratory and hypothesis-generating. Direct mechanistic and therapeutic relevance at the tissue level was not addressed by the present analysis and would require independent validation in prospective multi-center cohorts, alongside dedicated tissue-based and functional studies.

## Figures and Tables

**Table 1:** Number of patients in the two datasets.

Group		Number of Patients
Non-cancer		6245
Pan-cancer	biliary tract cancer	402
	bladder cancer	399
	bone and soft tissue sarcomas	299
	breast cancer	675
	colorectal cancer	1596
	esophageal squamous cell cancer	566
	gastric cancer	1418
	hepatocellular cancer	348
	intraparenchymal brain tumors	241
	lung cancer	1699
	ovarian cancer	400
	pancreatic cancer	851
	prostate cancer	1027
	Total	9921

**Table 2:** Performance of the classification models using the optimal feature subset and the inflection point subset in the pan-cancer and non-cancer comparative analysis.

Feature Ranking Algorithms	Classification Algorithm	Number of Features	SN	SP	Precision	F1 Measure	MCC	ACC
LASSO	KNN	25	0.979	0.965	0.946	0.962	0.938	0.970
LightGBM	KNN	25	0.985	0.983	0.973	0.979	0.965	0.983
MCFS	RF	15 *	0.919	0.992	0.986	0.952	0.924	0.964
	RF	645 **	0.940	0.991	0.985	0.962	0.940	0.971
mRMR	DT	50 *	0.961	0.962	0.941	0.951	0.919	0.962
	DT	175 **	0.966	0.966	0.946	0.956	0.928	0.966
RF_ZL	RF	25 *	0.928	0.985	0.976	0.951	0.923	0.963
	RF	995 **	0.945	0.993	0.989	0.966	0.947	0.975
CATboost	KNN	50	0.984	0.982	0.971	0.977	0.963	0.982
XGBoost	RF	35	0.962	0.987	0.979	0.970	0.952	0.977

Those with * markers are the number of features in the inflection point subset, and those with ** markers are the number of features in the optimal feature subset.

**Table 3:** Performance of the classification models using the optimal feature subset and the inflection point subset in thirteen cancer types comparative analysis.

Feature Ranking Algorithms	Classification Algorithm	Number of Features	Weighted F1	MCC	ACC
LASSO	SVM	60 *	0.783	0.766	0.789
	SVM	195 **	0.795	0.778	0.800
LightGBM	SVM	95 *	0.865	0.852	0.867
	SVM	245 **	0.884	0.873	0.886
MCFS	SVM	95 *	0.821	0.805	0.825
	SVM	480 **	0.869	0.857	0.871
mRMR	SVM	115 *	0.821	0.806	0.825
	SVM	615 **	0.850	0.837	0.853
RF_ZL	SVM	115 *	0.859	0.846	0.862
	SVM	295 **	0.876	0.865	0.878
CATboost	SVM	65 *	0.850	0.836	0.853
	SVM	155 **	0.878	0.866	0.880
XGBoost	SVM	140 *	0.821	0.805	0.825
	SVM	240 **	0.832	0.817	0.835

Those with * markers are the number of features in the inflection point subset, and those with ** markers are the number of features in the optimal feature subset.

**Table 4:** Performance of SVM model using top 245 features in the list yielded by LightGBM on thirteen cancer types.

Cancer Type	F1 Measure	Cancer Type	F1 Measure
biliary tract cancer	0.865	bladder cancer	0.951
bone and soft tissue sarcomas	0.949	breast cancer	0.926
colorectal cancer	0.767	esophageal squamous cell cancer	0.896
gastric cancer	0.884	hepatocellular cancer	0.915
intraparenchymal brain tumors	0.996	lung cancer	0.835
ovarian cancer	0.948	pancreatic cancer	0.933
prostate cancer	0.971	-	-

## Data Availability

Data analyzed in this study is available in GEO database with accession ID GSE211692 (https://www.ncbi.nlm.nih.gov/geo/query/acc.cgi?acc=GSE211692, accessed on 20 March 2023). The analyzed results are contained within the article or Supplementary Material.

## Supplementary Materials

The following supporting information can be downloaded at: https://www.mdpi.com/article/10.3390/life16050850/s1, Figure S1: IFS curves for the first analysis on pan-cancer and non-cancer populations comparison using the feature lists yielded by four feature-ranking algorithms. *y*-axis is the F1 measure metric and *x*-axis is the number of features. A. IFS curves based on the feature list yielded by LASSO. B. IFS curves based on the feature list yielded by LightGBM. C. IFS curves based on the feature list yielded by MCFS. D. IFS curves based on the feature list yielded by mRMR. Figure S2: IFS curves for the first analysis on pan-cancer and non-cancer populations comparison using the feature lists yielded by three feature-ranking algorithms. *y*-axis is the F1 measure metric and *x*-axis is the number of features. A. IFS curves based on the feature list yielded by RF_ZL. B. IFS curves based on the feature list yielded by CATboost. C. IFS curves based on the feature list yielded by XGBoost. Figure S3: IFS curves for the second analysis on patients with thirteen different cancer types using the feature lists yielded by four feature-ranking algorithms. *y*-axis is the Weighted F1 metric and *x*-axis is the number of features. A. IFS curves based on the feature list yielded by LASSO. B. IFS curves based on the feature list yielded by LightGBM. C. IFS curves based on the feature list yielded by MCFS. D. IFS curves based on the feature list yielded by mRMR. Figure S4: IFS curves for the second analysis on patients with thirteen different cancer types using the feature lists yielded by three feature-ranking algorithms. *y*-axis is the Weighted F1 metric and *x*-axis is the number of features. A. IFS curves based on the feature list yielded by RF_ZL. B. IFS curves based on the feature list yielded by CATboost. C. IFS curves based on the feature list yielded by XGBoost. Figure S5: Network degree plots of microRNA-genes network based on MIENTURNET platform and miRTarBase database (Pan-Cancer vs. non-Cancer). Figure S6: Violin plots showing the serum expression distributions of the nine miRNAs in Pan-Cancer versus Non-Cancer samples. Figure S7: Network degree plots of microRNA-genes network based on MIENTURNET platform and miRTarBase database (Within Pan-Cancer). Figure S8: Heatmap showing the class-specific expression distributions of the eight miRNAs across the thirteen solid cancer types. File S1: Description on feature-ranking and classification algorithms. Table S1. Details of machine learning algorithms used in this study. Table S2: Feature lists yielded by LASSO, LightGBM, MCFS, mRMR, RF_ZL, CATboost, XGBoost for two analyses. Table S3: Performance of the classification models constructed in the IFS procedure for the first analysis on pan-cancer vs. non-cancer. Table S4: Performance of the classification models constructed in the IFS procedure for the second analysis on thirteen cancer types. Table S5: Intersection of inflection point subsets identified from feature lists yielded by LASSO, LightGBM, MCFS, mRMR, RF_ZL, CATboost, XGBoost. The features that appear in 7, 6, 5, 4, 3, 2, and 1 inflection point subset are shown. Table S6: Classification rules generated by the optimal decision tree models on seven feature lists for the first analysis on pan-cancer vs. non-cancer. Table S7: Classification rules generated by the optimal decision tree models on seven feature lists for the second analysis on thirteen cancer types. Table S8: KEGG enrichment analysis results of miRNA-target network based on the miRNet platform for the first analysis on pan-cancer vs. non-cancer. Table S9: Description of genes related to the Pathways in Cancer and their distribution in functional sub-modules. Table S10: Expression statistics and ranking-algorithm selection for the miRNAs we selected. For each miRNA, the table reports mean ± SD and median [IQR] of log2-transformed serum expression in Non-Cancer and Pan-Cancer groups, log2 fold change (Pan-Cancer minus Non-Cancer), Wilcoxon rank-sum test *p* value with Benjamini–Hochberg correction (q), and the number of the seven ranking methods that selected the feature. Table S11: KEGG enrichment analysis results of miRNA-target network based on the miRNet platform for the second analysis on thirteen cancer types. Table S12: Shared miRNA target genes and their functions (within Pan-Cancer).

## Abbreviations

The following abbreviations are used in this manuscript:

miRNA	MicroRNA
CATboost	Categorical boosting
LASSO	Least absolute shrinkage and selection operator
LightGBM	Light gradient boosting machine
MCFS	Monte Carlo feature selection
mRMR	Minimum redundancy maximum relevance
XGBoost	Extreme gradient boosting
SMOTE	Synthetic minority oversampling technique
IFS	Incremental feature selection
RF	Random forest
KNN	k-nearest neighbor
DT	Decision tree
SVM	Support vector machine
LIFR	Leukemia inhibitory factor receptor
SFTPC	Surfactant protein C
LATS2	Large tumor suppressor 2
hBMSC	Human bone marrow mesenchymal stem cell
