# Supplementary material for: Machine Learning-Based Identification of Candidate Serum miRNA Features for Pan-Cancer and Cancer Type Classification
Source: Life (Basel). 2026 May 20;16(5):850. doi: 10.3390/life16050850 (PMC13208496; doi:10.3390/life16050850)
Supplement: Supplementary file 1 [file life-16-00850-s001.zip › life-4232501-supplementary/Figure S1.pdf]

A. IFS curves based on the feature list yielded by LASSO.

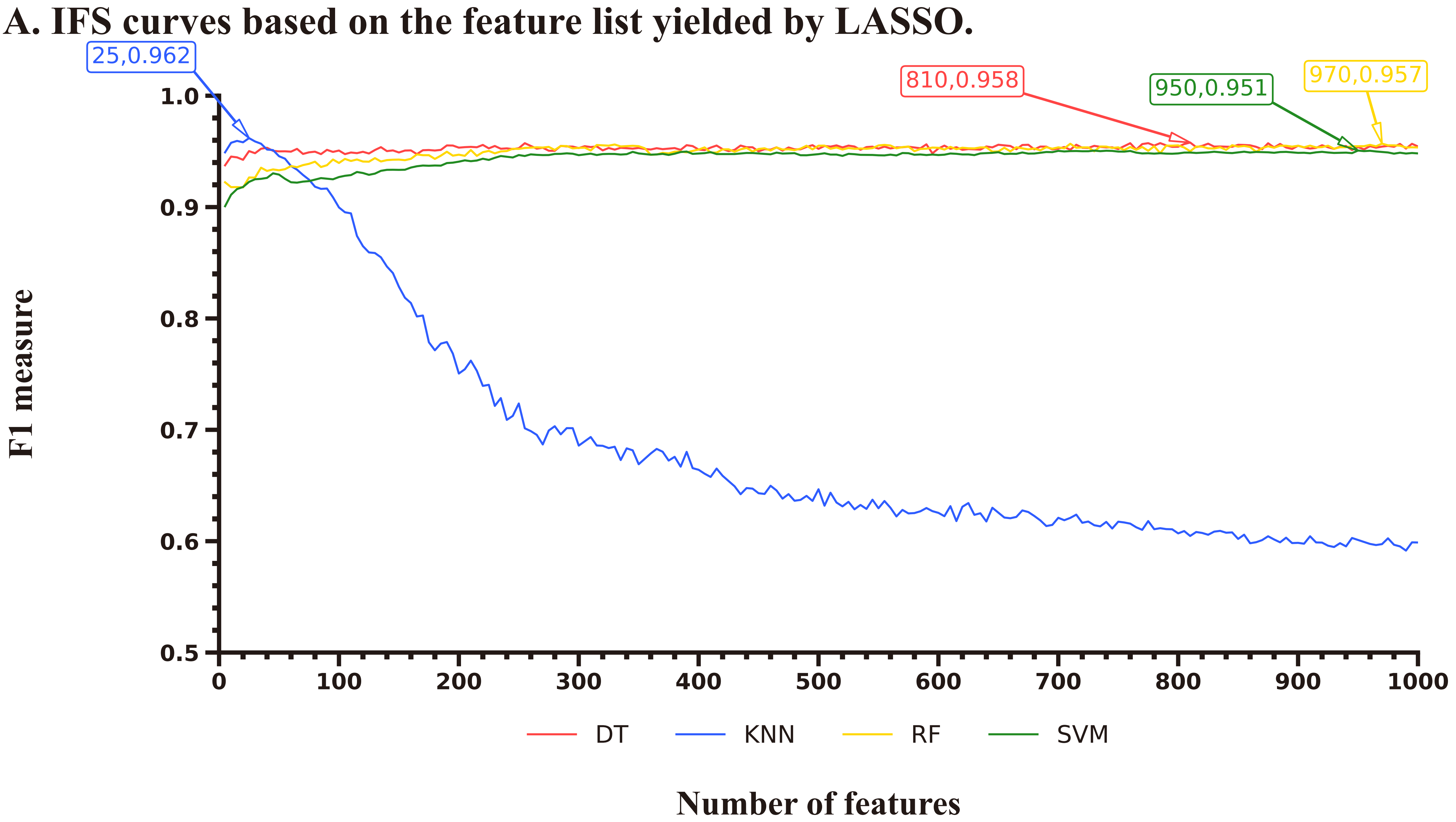

B. IFS curves based on the feature list yielded by LightGBM.

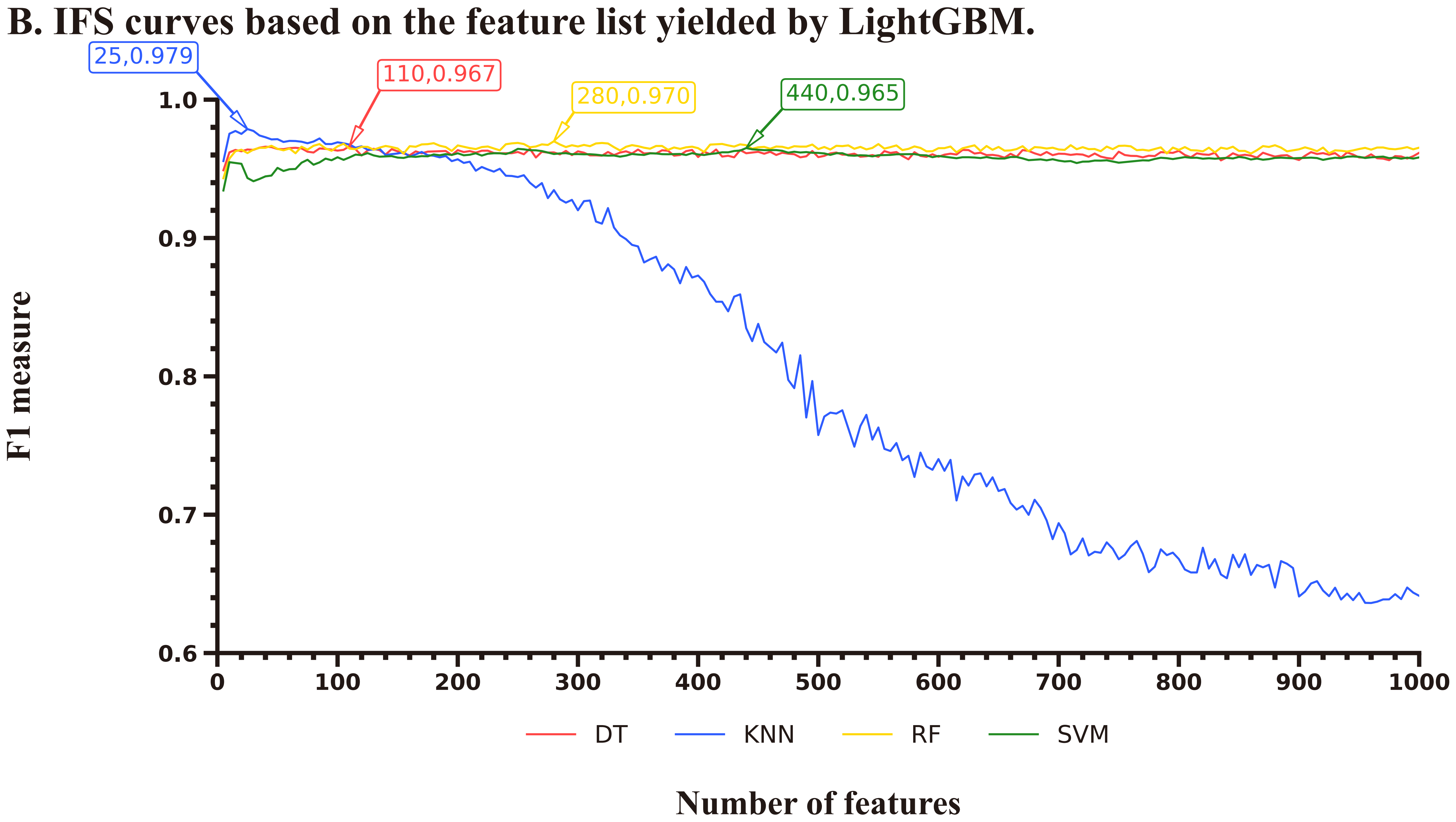

C. IFS curves based on the feature list yielded by MCFS.

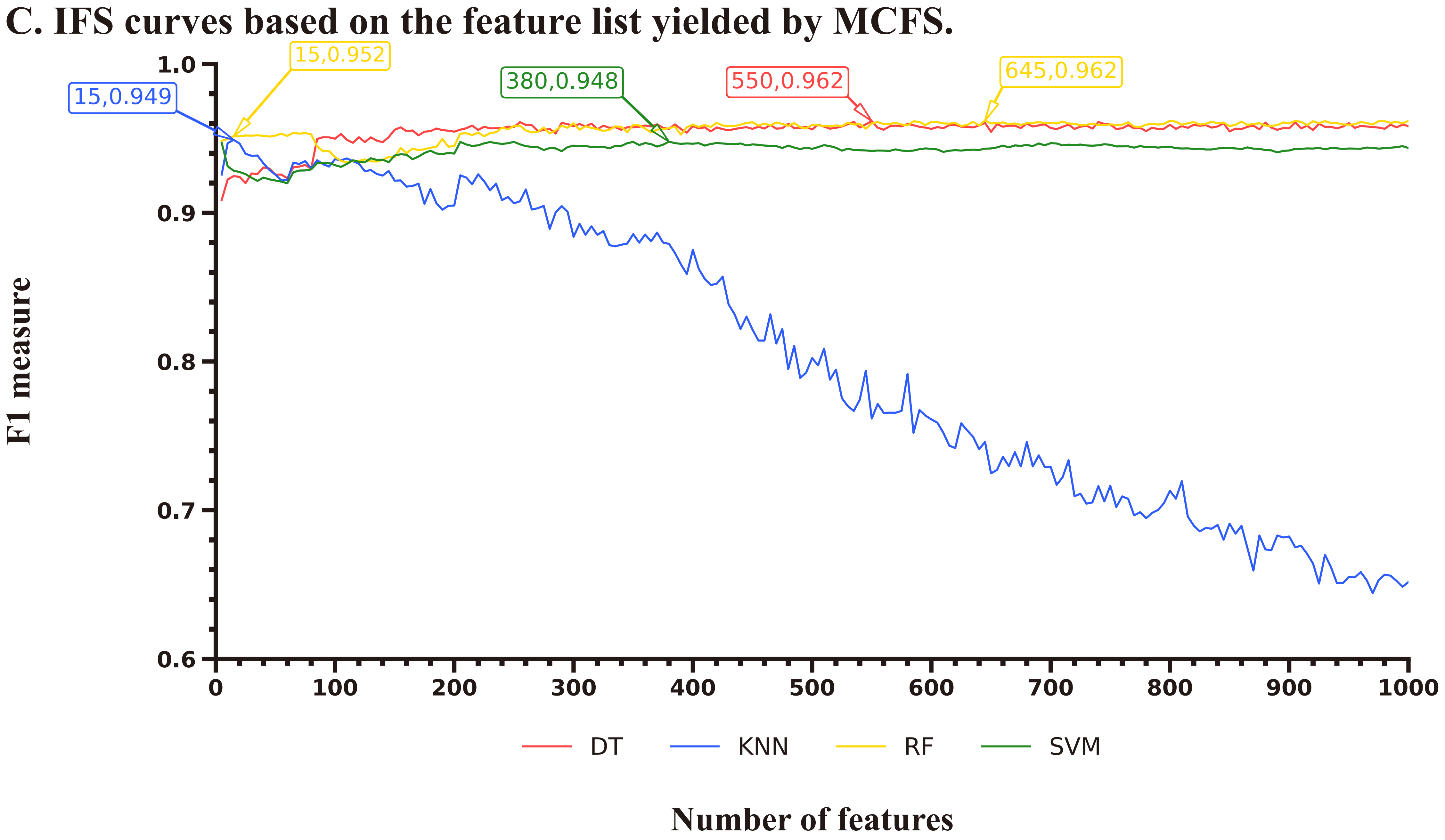

D. IFS curves based on the feature list yielded by mRMR.

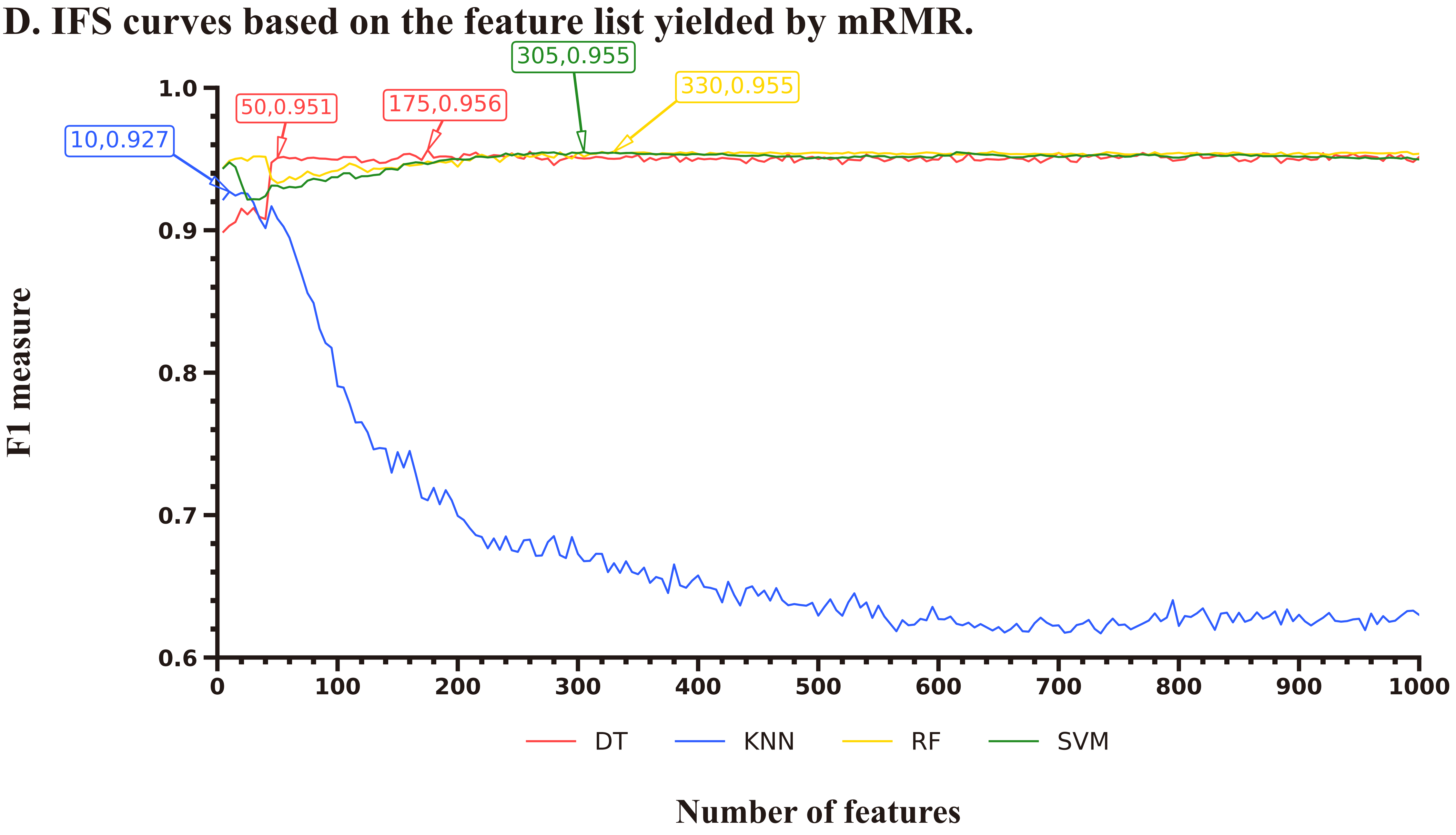

Figure S1: IFS curves for the first analysis on pan-cancer and non-cancer populations comparison using the feature lists yielded by four feature-ranking algorithms. y-axis is the F1 measure metric and x-axis is the number of features
