# Supplementary material for: Machine Learning-Based Identification of Candidate Serum miRNA Features for Pan-Cancer and Cancer Type Classification
Source: Life (Basel). 2026 May 20;16(5):850. doi: 10.3390/life16050850 (PMC13208496; doi:10.3390/life16050850)
Supplement: Supplementary file 1 [file life-16-00850-s001.zip › life-4232501-supplementary/Figure S2.pdf]

A. IFS curves based on the feature list yielded by RF_ZL.

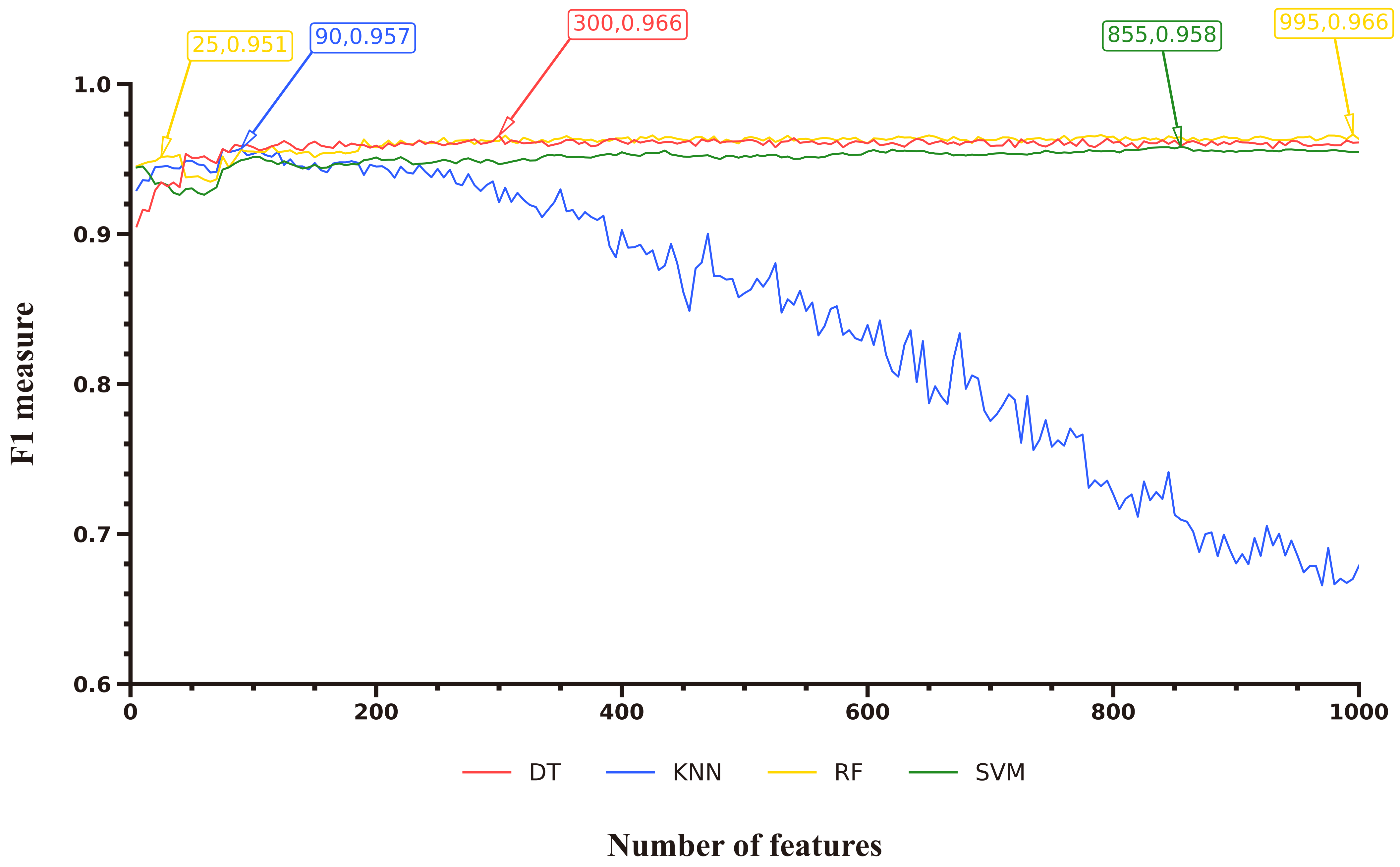

B. IFS curves based on the feature list yielded by CATboost.

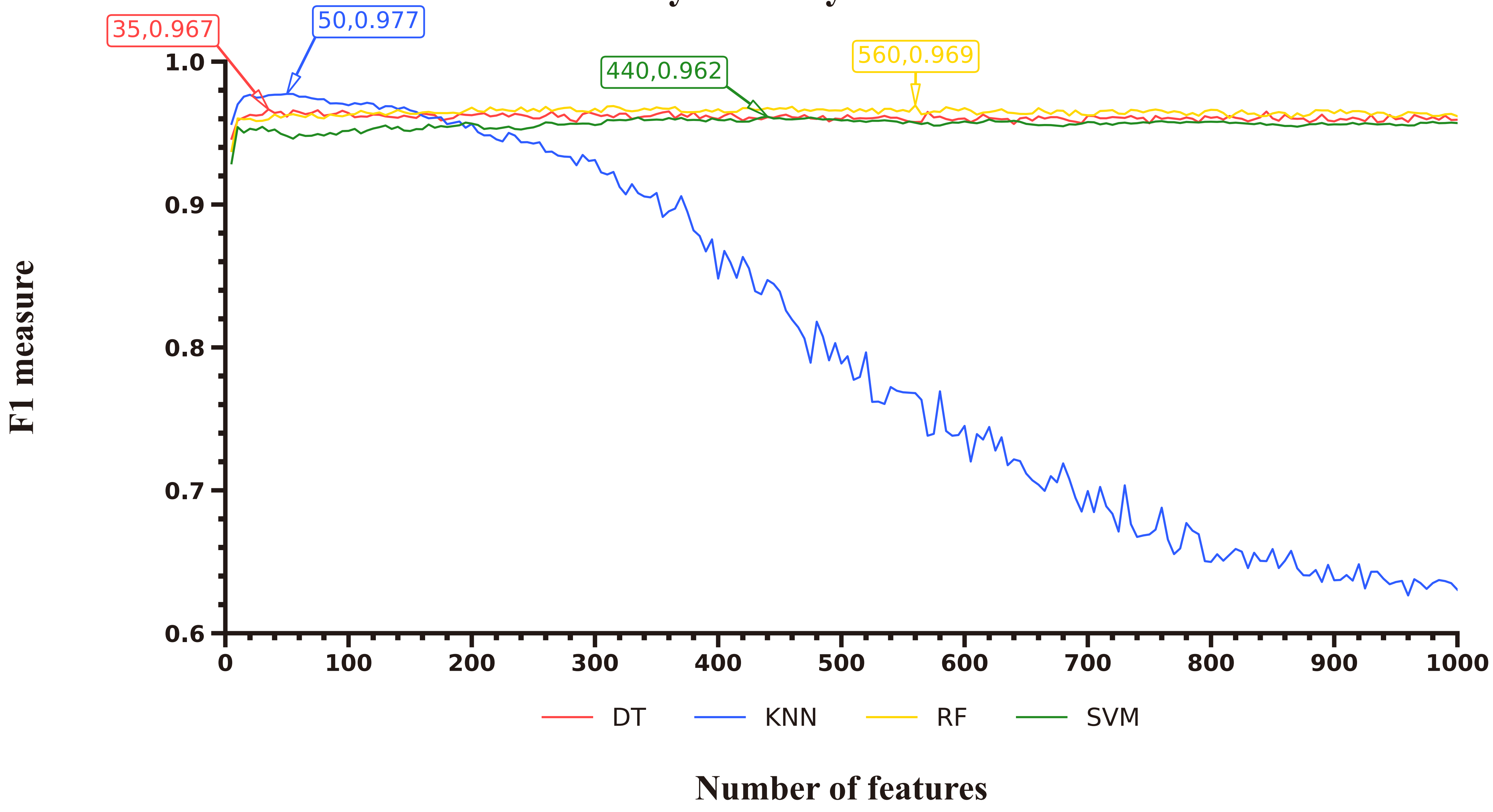

C. IFS curves based on the feature list yielded by XGBoost.

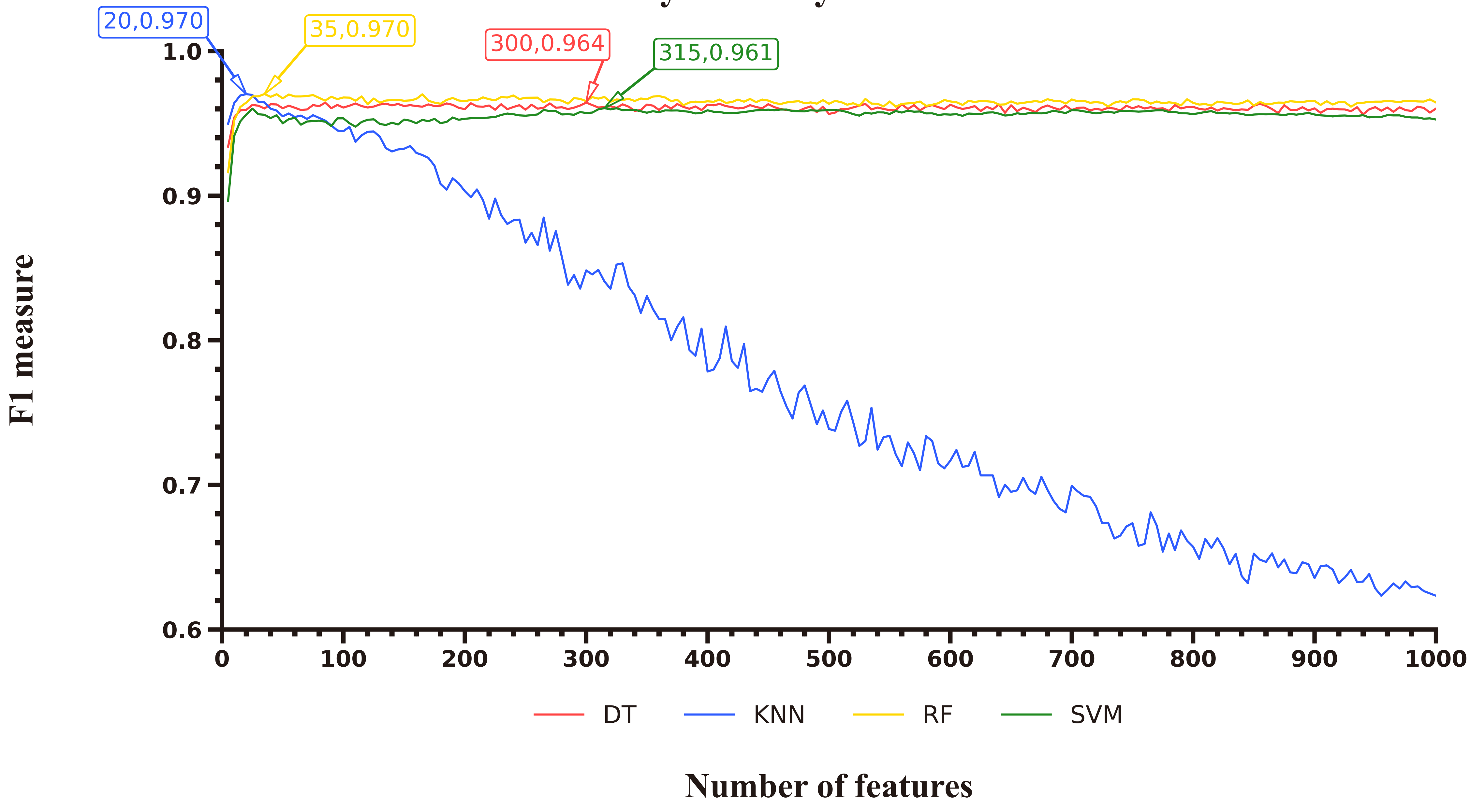

Figure S2: IIFS curves for the first analysis on pan-cancer and non-cancer populations comparison using the feature lists yielded by three feature-ranking algorithms. y-axis is the F1 measure metric and x-axis is the number of features.
