# Supplementary material for: Machine Learning-Based Identification of Candidate Serum miRNA Features for Pan-Cancer and Cancer Type Classification
Source: Life (Basel). 2026 May 20;16(5):850. doi: 10.3390/life16050850 (PMC13208496; doi:10.3390/life16050850)
Supplement: Supplementary file 1 [file life-16-00850-s001.zip › life-4232501-supplementary/Figure S5.pdf]

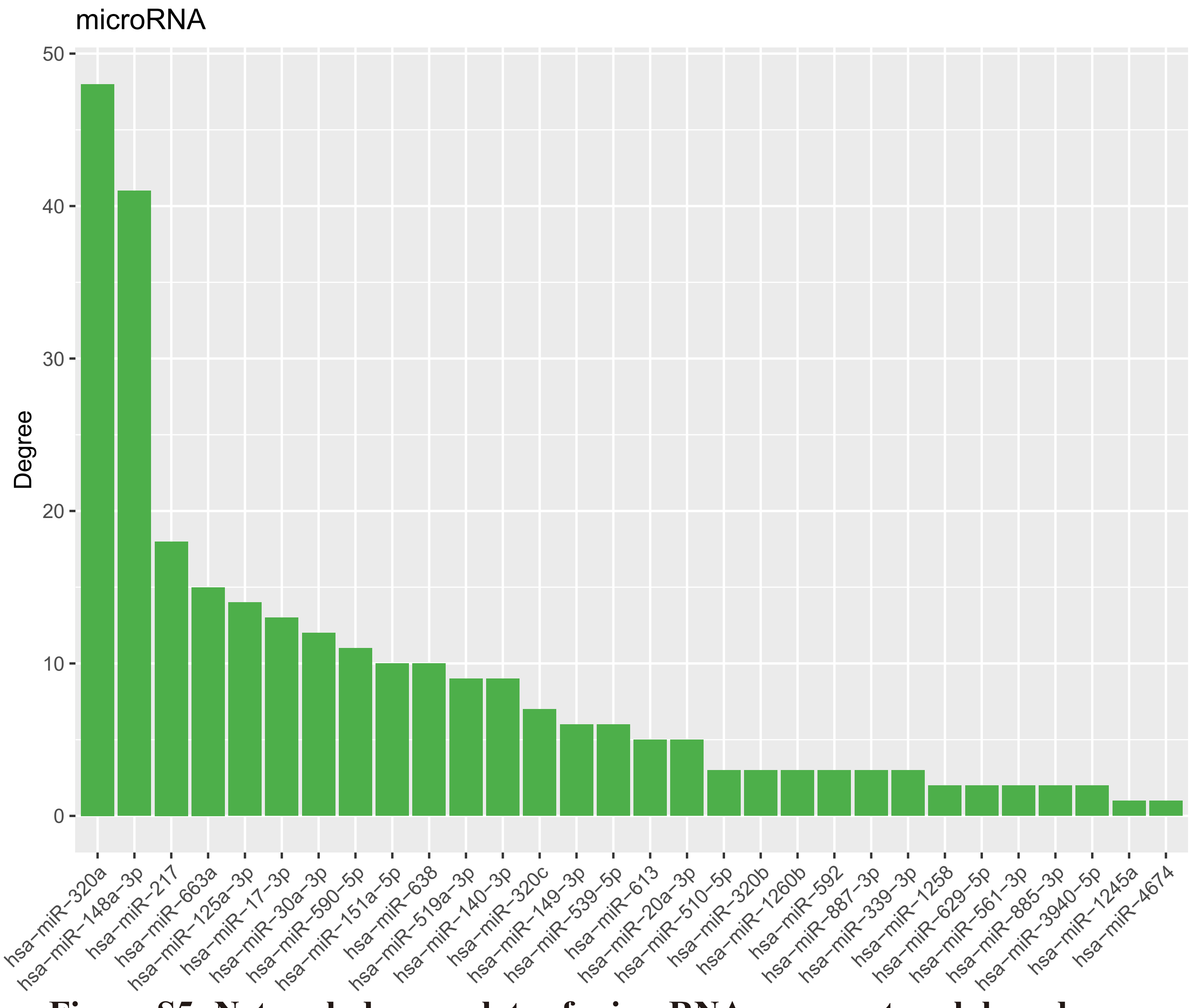

Figure S5: Network degree plots of microRNA-genes network based on MIENTURNET platform and miRTarBase database (Pan-Cancer vs non-Cancer).
