# Supplementary material for: Machine Learning-Based Identification of Candidate Serum miRNA Features for Pan-Cancer and Cancer Type Classification
Source: Life (Basel). 2026 May 20;16(5):850. doi: 10.3390/life16050850 (PMC13208496; doi:10.3390/life16050850)
Supplement: Supplementary file 1 [file life-16-00850-s001.zip › life-4232501-supplementary/File S1.pdf]

File S1. Description on feature-ranking and classification algorithms.

1. Feature-ranking algorithms

1.1 Least Absolute Shrinkage and Selection Operator

Least Absolute Shrinkage and Selection Operator (LASSO) [1] is a popular regression analysis method, which performs both variable selection and regularization. Lasso operates by introducing a penalty term into the loss function of the linear regression model, which is equal to the absolute value of the coefficient amplitude multiplied by a regularized parameter, usually expressed as λ. With the increase of this regularized term, more and more coefficients are reduced to zero, effectively "shrinking" them. When the coefficient is reduced to zero, it means that the corresponding features have been eliminated from the model. Features with non-zero coefficients after LASSO application are selected and considered as important features. Thus, by modulating the regularized term λ, LASSO is able to perform feature selection from feature pool.

1.2 Light Gradient Boosting Machine

Light Gradient Boosting Machine (LightGBM) is a gradient boosting framework developed by Microsoft that uses tree-based learning algorithms [2]. As a tree-based model, it can evaluate the importance of features in terms of prediction contribution. In evaluating the importance of features, two special methods are employed: 1. Importance of splitting: This method focuses on the frequency at which features act as splitting conditions in all decision trees. That is to say, the importance of a feature is evaluated by counting the number of times it is selected by the model. Those features that are often used in the decision-making process will naturally get higher split importance scores. 2. Importance of gain: this method focuses on the overall benefits brought by features in the process of splitting. The importance of features is evaluated by measuring their contribution to the improvement of accuracy when they participate in splitting. In essence, this method is to count the cumulative contribution of each feature to reducing the objective function in all the splits it participates in.

1.3 Monte Carlo Feature Selection

Monte Carlo Feature Selection (MCFS) [3] is a kind of popular methods to evaluate the importance of the features in a dataset. The core idea of MCFS lies in combining Monte Carlo sampling technology with decision tree algorithm to rank features. The operation process of this method can be summarized as the following three stages: 1. Monte Carlo sampling stage: in this initial stage, MCFS constructs a number of different feature subsets by repeatedly sampling randomly from the data set. Then, for each feature subset, the system will generate several training data subsets through random sampling. 2. Decision tree learning stage: the system will process each generated training data subset and train the corresponding decision tree respectively. 3. Feature importance evaluation stage: after all decision trees are established, the system begins to evaluate the importance of each feature. The evaluation process mainly considers three aspects: the weighted accuracy of each decision tree, the information gain, and the coverage of features in each node of the tree.

1.4 Minimum Redundancy Maximum Relevance (mRMR)

Minimum Redundancy Maximum Relevance (mRMR) is a feature selection method that aims to select features that are more correlated with target variables but less correlated with each other [4]. The method operates on two basic principles, in

which the principle of maximum relevance focuses on selecting features with high relevance to target class or label, while the principle of minimum redundancy is devoted to minimizing the correlation between selected features. The measurement of correlation is based on by mutual information theory. In the implementation, The mRMR iteratively chooses the features that maximize the relevance with target variable and minimize the correlation between feature step-by-step until a specified number of features are collected into the selection feature set.

1.5 Random Forest

Random forest (RF_ZL) [5] is a kind of popular tree-based models. The idea of RF is to estimate the importance of features by randomly changing feature values and measuring the degradation of resulting model performance. RF provides a natural way to measure the importance of features. In the context of random forests, feature importance is usually calculated based on "Gini importance/ Mean Decrease Impurity " or " Permutation importance". 1. Gini Importance / Mean Decrease Impurity: this is the total reduction of average node impurities of all trees in the set. More simply, it calculates the contribution of each feature to the homogeneity of nodes and leaves in the generated random forest model. The higher the value, the more important the feature is. 2. Permutation importance: This is a more direct measure of the impact on accuracy by randomly permutating the feature position in the tree-based learning structures.

1.6 Categorical Boosting

Categorical Boosting (CATboost) [6] developed by Yandex is a high-performance open-source library, which is used for gradient boosting on decision trees. The name CATboost comes from two words' category' and' boosting'. Notably, it is designed to handle categorical features rather than numerical features. CATboost provides state-of-the-art accuracy for various tasks. Its performance is equivalent to other gradient boosting algorithms, even better than those gradient boosting algorithms on utilizing categorical features. Specially, CATboost is less sensitive to setting of hyperparameters, which means it could provide good and robust performance even with default parameters. CATboost also provides a built-in feature importance evaluation method, which is similar to the Mean Decrease Impurity method used in other tree-based algorithms, and it is evaluated by measuring the number of times each feature is used as a data split point in all decision trees and how much the feature contribute to the improvement of model performance.

1.7 Extreme Gradient Boosting

Extreme Gradient Boosting (XGBoost) [7], as an open-source software library providing gradient boosting framework, is famous for its excellent performance and computation efficiency, which make it a widely used tool in machine learning competitions and industrial. The importance of features in XGBoost can be calculated and explained by using the following methods: 1. Split method, which measures the importance by calculating the frequency of features used for data splitting in all decision trees, that us, the higher the frequency of use, the greater the importance; 2. Gain method, which evaluates the contribution of each feature by measuring its average gain in the decision tree, specifically calculating the contribution of each feature to the overall reduction of the loss function, that is, the higher the gain value, the greater the importance of the feature. 3. Coverage method, which pays attention to the average coverage of features in the decision tree, and determines its importance by calculating the number of samples affected by the splits based on a feature which reflecting the influence range of features on the data set.

2. Classification Algorithm

2.6.1 Decision Tree

Decision tree (DT) is a powerful machine learning algorithm, which is widely used in data mining and predictive modeling [8]. Its core idea is to decompose a complex decision-making process into a series of simpler decisions, thus improving the interpretability and predictive ability of the model. The structure of the decision tree model is similar to the flow chart, showing a tree-like hierarchical structure, in which the top root node is responsible for dividing the data into multiple subsets based on a certain feature or attribute, and the branches formed by these divisions are either connected to the decision nodes that are further divided by other unused features, or terminated at the leaf nodes (also called terminal nodes) that cannot be further subdivided, and these leaf nodes represent the final prediction or decision results of the model.

2.6.2 k-Nearest Neighbors

K-Nearest Neighbor algorithm (KNN) [9] is a simple and easy-to-implement supervised machine learning algorithm. Its basic principle is to predict the labels of target data points by observing the' k' nearest marked data points in feature space. The implementation process of this algorithm first needs to determine a user-defined constant' k', which determines the number of neighbors participating in the voting, and then calculate the distance between data points by using distance measurements such as Euclidean distance, Manhattan distance or Minkowski distance. After identifying the' k' observation values closest to a specific test point in the training data, the algorithm determines the classification of the target object through the majority voting of the neighbors.

2.6.3 Random Forest

A Random Forest (RF) [5] is a kind of popular tree-based models. The idea of RF is to estimate the importance of features by randomly changing feature values and measuring the degradation of resulting model performance. RF provides a natural way to measure the importance of features. In the context of random forests, feature importance is usually calculated based on "Gini importance/ Mean Decrease Impurity " or " Permutation importance". 1. Gini Importance / Mean Decrease Impurity: this is the total reduction of average node impurities of all trees in the set. More simply, it calculates the contribution of each feature to the homogeneity of nodes and leaves in the generated random forest model. The higher the value, the more important the feature is. 2. Permutation importance: This is a more direct measure of the impact on accuracy by randomly permutating the feature position in the tree-based learning structures.

2.6.4 Support Vector Machine

The principle of Support Vector Machine (SVM) [10] is to find a hyperplane that separates the data in such a way that the margin between the hyperplane and the closest points from each class is maximized in the latent space. These closest points are called support vectors, and they are the critical elements of the dataset as they define the hyperplane, that is, the decision boundaries among classes in the latent space. SVMs can handle both linear and non-linear data. For linear data, a linear boundary can separate the data. If the data is not linearly separable, the SVMs can construct high-dimensional hyperplane by using a special mathematical tool called as "kernel trick". The "kernel trick" transformation maps the input space into a higher-dimensional space where the data could be linearly separable. SVMs have a regularization parameter,

usually denoted by 'C' in equations, which helps make a trade-off between a low training error and minimization of model complexity, that is, to control the width of hyperplane in latent space. A smaller 'C' will result in a wider margin and might misclassify more points, thus giving a simpler model that will underfit the data. A higher 'C' gives a narrower margin, risking overfitting the data.

References

1. Ranstam, J.; Cook, J.A. Lasso regression. *British Journal of Surgery* **2018**, *105*, 1348-1348.
2. Ke, G.; Meng, Q.; Finley, T.; Wang, T.; Chen, W.; Ma, W.; Ye, Q.; Liu, T.-Y. In *Lightgbm: A highly efficient gradient boosting decision tree*, Guyon, I.; Luxburg, U.V.; Bengio, S.; Wallach, H.; Fergus, R.; Vishwanathan, S.; Garnett, R., Eds. Curran Associates, Inc.: 2017///.
3. Dramiński, M.; Koronacki, J. Rmcfs: An r package for monte carlo feature selection and interdependency discovery. *Journal of Statistical Software* **2018**, *85*, 1 - 28.
4. Peng, H.; Long, F.; Ding, C. Feature selection based on mutual information: Criteria of max-dependency, max-relevance, and min-redundancy. *IEEE Transactions on Pattern Analysis and Machine Intelligence* **2005**, *27*, 1226-1238.
5. Breiman, L. Random forests. *Machine learning* **2001**, *45*, 5-32.
6. Dorogush, A.V.; Ershov, V.; Gulin, A. Catboost: Gradient boosting with categorical features support. *arXiv preprint arXiv:1810.11363* **2018**.
7. Chen, T.; Guestrin, C. In *Xgboost: A scalable tree boosting system*, The 22nd ACM SIGKDD International Conference on Knowledge Discovery and Data Mining, San Francisco, CA, USA, 13-17 August 2016, 2016; Association for Computing Machinery: San Francisco, CA, USA, 2016; pp 785-794.
8. Safavian, S.R.; Landgrebe, D. A survey of decision tree classifier methodology. *IEEE transactions on systems, man, and cybernetics* **1991**, *21*, 660-674.
9. Cover, T.; Hart, P. Nearest neighbor pattern classification. *IEEE Transactions on Information Theory* **1967**, *13*, 21-27.
10. Cortes, C.; Vapnik, V. Support-vector networks. *Machine Learning* **1995**, *20*, 273-297.
