# Supplementary material for: Machine Learning-Based Identification of Candidate Serum miRNA Features for Pan-Cancer and Cancer Type Classification
Source: Life (Basel). 2026 May 20;16(5):850. doi: 10.3390/life16050850 (PMC13208496; doi:10.3390/life16050850)
Supplement: Supplementary file 1 [file life-16-00850-s001.zip › life-4232501-supplementary/Table S1.pdf]

Table S1. Details of machine learning algorithms used in this study.

Algorithm	Functions in this study	URL or platform
Least absolute shrinkage and selection operator	Feature ranking	Scikit-learn
Light gradient boosting machine	Feature ranking	https://lightgbm.readthedocs.io/en/latest/
Monte Carlo feature selection	Feature ranking	http://www.ipipan.eu/staff/m.draminski/mcfs.html
Minimum redundancy maximum relevance	Feature ranking	http://home.penglab.com/proj/mRMR/
Random forest	Feature ranking, Classification	Scikit-learn
Categorical boosting	Feature ranking	https://catboost.ai/en/docs/concepts/installation
Extreme gradient boosting	Feature ranking	https://xgboost.readthedocs.io/en/stable/
Decision tree	Classification	Scikit-learn
K-nearest neighbors	Classification	Scikit-learn
Support vector machine	Classification	Scikit-learn
Synthetic Minority Over-sampling Technique	Dataset balanced	https://github.com/scikitlearn-contrib/imbalanced-learn
