# Supplementary figures and images for: Machine Learning-Based Identification of Candidate Serum miRNA Features for Pan-Cancer and Cancer Type Classification
Source: Life (Basel). 2026 May 20;16(5):850. doi: 10.3390/life16050850 (PMC13208496; doi:10.3390/life16050850)

Heatmap of Median Expression across Cancer Types  
(Z-score normalized per miRNA; 13 solid tumors, n=9,921)

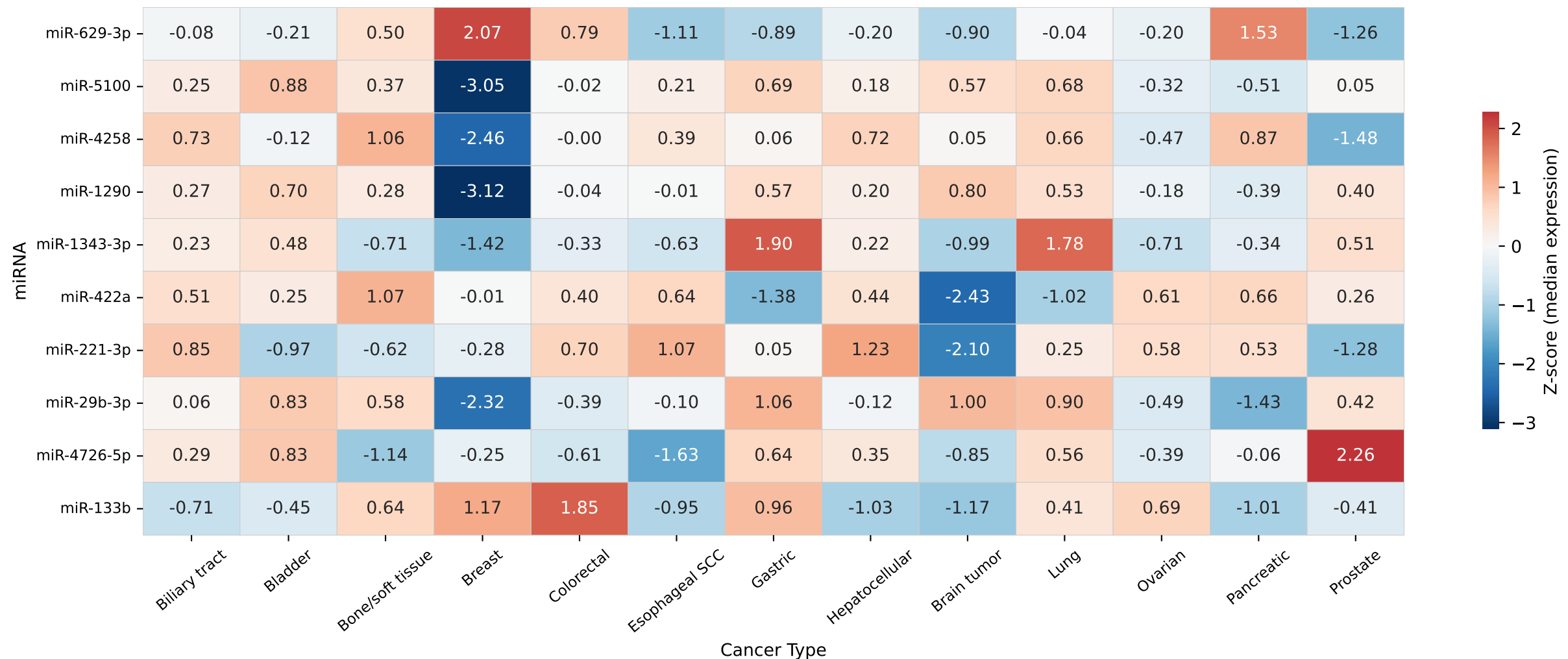

Supplement: Supplementary file 1 [file life-16-00850-s001.zip › life-4232501-supplementary/Figure S8.pdf]
